# Supplementary material for: Musical Scents: On the Surprising Absence of Scented Musical/Auditory Events, Entertainments, and Experiences
Source: Iperception. 2021 Sep 23;12(5):20416695211038747. doi: 10.1177/20416695211038747 (PMC8474342; doi:10.1177/20416695211038747)
Supplement: sj-pdf-1-ipe-10.1177_20416695211038747 - Supplemental material for Musical Scents: On the Surprising Absence of Scented Musical/Auditory Events, Entertainments, and Experiences [file sj-pdf-1-ipe-10.1177_20416695211038747.pdf]

RUNNING HEAD: SOUNDS AND SCENTS

Musical scents: On the surprising absence of scented musical/auditory events, entertainments, and experiences

Prof. Charles Spence,

Head of the Crossmodal Research Laboratory, University of Oxford

RESUMBITTED TO: *i-Perception* (Our Special Sense of Smell)

WORD COUNT: 16,750 WORDS

DATE: JULY, 2021

CORRESPONDENCE TO: Prof. Charles Spence, Department of Experimental Psychology, Anna Watts Building, University of Oxford, Oxford, OX2 6BW, UK.

EMAIL: charles.spence@psy.ox.ac.uk.

ABSTRACT

The matching of scents with music is both one of the most natural (or intuitive) of crossmodal correspondences and, at the same time, one of the least frequently explored combinations of senses in an entertainment and multisensory experiential design context. This narrative review highlights the various occasions over the last century or two when scents and sounds have coincided, and the various motivations behind those who have chosen to bring these senses together: This has included everything from the masking of malodour to the matching of the semantic meaning or arousal potential of the two senses, through to the longstanding and recently-reemerging interest in the crossmodal correspondences (now that they have been distinguished from the superficially similar phenomenon of synaesthesia, with which they were previously often confused). As such, there exist a number of ways in which these two senses can be incorporated into meaningful multisensory experiences that can potentially resonate with the public. Having explored the deliberate combination of scent and music (or sound) in everything from 'scent-sory' marketing through to fragrant discos and olfactory storytelling, I end by summarizing some of the opportunities around translating such unusual multisensory experiences from the public to the private sphere. This will likely be via the widespread dissemination of sensory apps that promise to convert (or translate) from one sense (likely scent) to another (e.g., music), as has, for example already started to occur in the world of music selections to match the flavour of specific wines.

KEYWORDS: MUSIC; SCENT; EXPERIENTIAL MARKETING; MULTISENSORY EXPERIENCE DESIGN; SOUND.

1. Introduction

Across the arts, there has been a growing awareness in recent years of the importance of delivering experiences that attract the public, especially as the pressure has mounted on many public bodies to justify any taxpayer funding they receive (Carù & Cova, 2005). Very often, the urge has been to try and engage more of the audience's/visitor's senses by putting-on multisensory experiential events (think here only of the popular appeal of classical music concerts paired with fireworks, as but one example). The push to engage better with the general public, and thus to increase audience numbers, has been felt especially strongly by those in the world of classical music where attendance figures, while never especially high, have been declining for years now. This has led to calls from at least one prominent conductor in the UK, for urgent reform in order to try and stay relevant – the suggestion being that symphony orchestras need to 'Jazz it up' if they are to survive (e.g., Sanderson, 2018).

Given such a backdrop, it is natural to consider whether adding scent might offer one route to enlivening music concerts (no matter what the type of music). After all, there is a long history of scents having been incorporated into live-performance settings (e.g., McGinley & McGinley, 2018; Reynolds, 1989; and see Spence, 2021a, for a recent review), though still falling some considerable way short of the 30 plus scents that were, on occasion, released into movie theatres in the brief heyday of scented cinema (see Spence, 2020d, for a review). At the same time, however, it is important to remember that we are visually-dominant creatures (Hutmacher, 2019), and that the relevance/importance of scent has long been downplayed (McGann, 2017). Furthermore, it is also worth stressing that the addition of an olfactory component to various theatrical, operatic, and cinematic entertainments has not always been deemed a success (see Spence, 2020d, 2021a, for reviews). Indeed, no matter whether one is talking about the theatre or the cinema, the incorporation of scent has sometimes been dismissed by commentators as nothing more than a gimmick (e.g., Gilbert, 2008, p. 167; Reynolds, 1989; Marks, 1999; see Spence, in press-a). Moreover, in large spaces, such as opera houses or, presumably, also many concert halls, it can be difficult to control the dispersal of scent. Such spaces are, remember, typically optimized for the transmission of sound, not scent.

One solution to the latter problem, namely of how to disperse the scent to all members of the audience, has involved the use of scratch and sniff cards, as in the 1989 production of Prokofiev's opera, *Love for Three Oranges* in London (Reynolds, 1989). In this case, the scratch and sniff cards were also distributed to those watching at home via a popular printed

national TV guide. According to reviews from those who had the opportunity to 'enjoy' this olfactorily-enhanced audiovisual entertainment, the scents were not presented at moments of dramatic tension, but rather when there was lots of noise on stage. As Robert Maycock put it: *"Musically the performance has an intensity to match the ...and carry the horrors of the scratch-and-sniff cards which fill the house with vaguely chemical odours and double the coughing score of an already seasonally bronchitic audience"* (Maycock, 1989). One of the more unpleasant odours used for this particular performance was described as smelling like *'a cross between bad eggs and body odour'* (Reynolds, 1989).[1] This, then, just one attempt of many to bring a scented component to a live performance setting. At the same time, however, there is also a danger that the different sensory inputs may compete for the audience's attention (see McGinley & McGinley, 2018, p. 222). And, what is more, having people scratch and sniff (typically in response to a numerical visual prompt) can also interrupt the natural flow of the entertainment experience.

If one looks back over the last century or two, it is striking how many attempts there have been to augment a wide variety of public entertainment formats by means of the addition of scent. Most famously, perhaps, have been the attempts to add scent to cinema in the middle decades of the 20th Century – think Smell-O-Vision, AromaRama, and subsequently Odorama. The question to be addressed here is why, in contrast, there would seem to have been so few attempts to bring an olfactory element to public music events, be it classic music or pop concerts, or even discos/nightclubs? And, beyond that, given the interest in adding scent to home TV, video-gaming, and virtual reality (VR) (see Spence, in press-a, for a recent review), one might also wonder why there have been so few attempts to connect, and ideally to enhance, the public's experience of home/personal multisensory music listening by means of 'scent-sory' augmentation? After all, listening to music presumably constitutes one of the most popular forms of home entertainment (Bull, 2000; Bull & Black, 2005; North & Hargreaves, 2008; Persolaise, 2020). The apparent absence of scented music is surprising inasmuch as there are various reasons to consider this pair of senses (namely, audition and olfaction) as matching particularly well and/or likely to offer successful pairings.

---

[1] Intriguingly, the results of laboratory-based VR studies suggest that bad smells tend to lead to greater immersion in virtual environments than either positive or neutral smells (e.g., Baus & Bouchard; 2017; Ischer, Baron, Mermoud, Cayeux, Porcherot, Sander, & Delplanque, 2014). The same may well also be true in a live-performance setting.

In this review, I will look at the history of public pairing, or combining, of scent and sound. This comprises everything from listening to live music concerts, gigs, and well as listening to digital music while at home, while on the move or else while shopping. I analyse the various reasons that have lain behind scented musical events. Given the widespread and historic interest in adding scent to everything from theatre performances to opera (Spence, 2021a), and from the cinema and TV (Spence, 2020d, in press-b) through to art galleries and museums (Spence, 2020c), it is perhaps surprising that olfactorily-enhanced home/personal musical experiences have yet to attain anything like the same popularity. Indeed, to date, there have been only very limited forays into the world of scented music outside of the nascent space of multisensory experiential/performance (see Di Stefano, Murari, & Spence, 2021, for a review). Finally, in this review, I will also consider how, in the future, such scented musical experiences, or more likely musical scent experiences, may be brought into the home/personal environment by means of digital sensory apps.

Given that the theatre, the cinema, and the opera have all tried, at various times, to introduce scent, it is then perhaps surprising that there have been so few examples of scent-enabled musical performances at least that have been described in the literature. This may potentially link to the different uses to which scent has been put (Banes, 2001). Note also that the introduction of scent to other entertainment formats argues that its neglect in the specific context of musical events cannot simply be put down to the longstanding downplaying of the sense of smell amongst humans (McGann, 2017). Here, at the outset, one might wonder whether scent is primarily used in an entertainment context to help support a narrative and/or even to tell stories rather than necessarily to induce, or convey, a particular mood, and it is the latter that is a somewhat more common feature of our experience of listening to music.

2. On the close connections between music and scent

A priori, there would seem to be a number of reasons to believe that the coordinated delivery of music and scent would be more popular than, in fact, it appears to have been. For one thing, the language used to describe both music and scent are, in a number of cases, closely shared (see Deroy, Crisinel, & Spence, 2013; Nightingale, 2020a; though see also Yudov, n.d.). Think here only of how terms such as ‘low/bass notes’, ‘high/top notes’, ‘chords’, and ‘harmonies’ can all be used to describe both music and fragrance. Further supporting the similarity, when

Sophia Grojsman, a Belarusian perfumer, was interviewed by Diane Ackerman (1990), she suggested that composing music was similar to producing a fragrance.

Furthermore, going back to the middle of the 19th Century, one finds Septimus Piesse (1862/1891) famously putting forward his scent scale, in which he matched 24 musical notes to a range of scents (see **Figure 1**).[2] In his treatise, Piesse explicitly noted how sounds and odours blend together similarly, producing different degrees of "a nearly similar impression" in the sensory nerves (Piesse 1867, p. 39). Piesse also writes about how the mixture needed to prepare the odours for the handkerchief evokes effects on the smelling nerve "similar to that which music or the mixture of harmonious sounds produces upon the nerve of hearing, that of pleasure" (Piesse 1867, p. 219). Remarkably, Piesse even states that creating a mixture of scents is like creating a mixture of sounds, i.e., chords, writing at one point that: "We have citron, lemon, orange peel, and verbena, forming a higher octave of smells, which blend in a similar manner" (Piesse 1867, p. 39). Piesse was seemingly convinced that the pleasantness of musical harmony[3] resembles that of perfumes (consisting of various base notes), and he presented a scale of correspondences between sounds and odours, as he believed that "there is, as it were, an octave of odours like an octave in music" (Piesse 1867, p. 38).

INSERT FIGURE 1 ABOUT HERE

Some commentators have suggested that Piesse's scent scale constitutes one of the earliest attempts to establish systematic crossmodal correspondences[4] (at least between this particular pair of sensory modalities; e.g., see Deroy et al., 2013). However, a closer reading of Piesse's work, would rather appear to suggest that the perfumer's main point was not so much to stress the one-to-one correspondence between musical notes and scents as it was to draw attention to the way in which certain combinations of musical notes can be combined to give rise to a harmonious sound in much the same way that specific scents can also be combined to deliver

---

[2] Note that there is also some mention of scent and music being linked in the ancient Chinese Confucian ritual classics (see Lam, 1994).

[3] It is an interesting empirical question as to whether olfactory harmony works in the same way as in the auditory modality (see Athanasopoulos, Eerola, Lahdelma, & Kaliakatsos-Papakostas, 2021; cf. Cooke & Myin, 2011).

[4] Crossmodal correspondences have been defined as the sometimes surprising associations that people experience between seemingly-unrelated features, attributes, or dimensions of experience in different sensory modalities (see Spence, 2011). Over the years, they have often been confused with synaesthesia (Fakotakis, 2018; see Deroy & Spence, 2013, for a review).

a pleasing (and harmonious) olfactory combination.[5] One might though perhaps say that Piesse's interest was more on intramodal, than on crossmodal, perceptual grouping (see Spence, 2015a).[6]

A little over a century ago, Hartmann (1913) argued that the relation of scents to corresponding notes in Piesse's scent scales was *'based purely on individual opinion.'* A few years ago, here at the Crossmodal Research Laboratory, we attempted to revisit Piesse's scent scale in order to assess the consensuality of the crossmodal mapping a century and a half after these innovative mappings had first been published. Unfortunately, however, our results were inconclusive. That said, one point to note here is that musical scales (or rather the specific auditory frequencies that are associated with different musical notes) only became standardized around 1855 (i.e., the year before Piesse's work first appeared in print; see Kettler, 2015) thus making any straightforward assessment a little more challenging (pers. comm. William McVicker, organ builder). As such, one might wonder whether the 'Perfumery Organ, 2015/17', that was displayed at the NTT Intercommunication Centre in Tokyo, Japan to recreate Piesse's Gamut of Odours, used exactly the same mapping of scents to sounds as Piesse had originally had in mind.

Taking the analogy between sound and scent even further (though some might say too far), Aldous Huxley famously wrote about the scent organ in his novel *Brave New World*: *'The scent organ was playing a delightfully refreshing Herbal Capriccio – rippling arpeggios of thyme and lavender, of rosemary, basil, myrtle, tarragon; a series of daring modulations through the spice keys into ambergris: and a slow return through sandalwood, camphor, cedar and new-mown hay (with occasional subtle touches of discord – a whiff of kidney pudding, the faintest suspicion of pig dung) back to the simple aromatics with which the piece began.'* (Huxley,

[5] At the same time, however, it is worth noting that there is also an intriguing separate literature amongst those who have wanted to match colours (hue) with musical notes (Caivano, 1994; Newton, 1954; Pridmore, 1992; Wells, 1985). However, in the latter case, it would seem that the motivation behind these various attempts, none of which, it should be said, has received widespread acceptance, has been based more on the structural relations between the organization of the two senses (Schöffer, 1985; Sebba, 1991), than necessarily on any strong perceptual affinity (or similarity).

[6] Camille Saint-Saëns, the celebrated French composer wrote a mini-extravaganza for an orchestra of toy instruments including two trumpets, harp, piano, and strings titled *Les Odeurs de Paris* that was published in 1871. However, there is little connection to olfaction in this work other than the title. In 2008, there was competition (called The Sound of Perfume) at the Royal College of Music to create a composition to match the scent of Clive Christian No. 1

1932, pp. 198-199). Huxley's fictional account would appear to be based on a transposition of music to a scented medium.

In a 2013 project going by the name of "Essence in Space", engineer Chang Hee Lee constructed an adapted keyboard that linked sound and fragrance to create a unique perfume, what Lee calls a 'Symphonic Perfume' (see Lee, 2013). Each musical key was mechanically linked to a specific fragrance that was situated below the keyboard so that when a key was depressed, a droplet of perfume was released and collected in a bottle. In this case, the 12 fragrances were based on Michel Edwards 12 fragrance categories (Edwards, 1984). Lower notes were paired with woody and floral scents, while higher musical notes were paired with fresh and watery scents instead (see **Figure 2**).[7] This process continued as each key was struck, resulting in a mixture of different droplets of perfume collecting in the bottle. At the end of the 'performance', a unique perfume is created. This work again draws attention to the link between sound and scent, with Lee referencing synaesthesia, and talking of a 'mystic identity'. On the website, Lee (2013) continues: "This mystic identity is produced through the transformation of sound and scent. As a form of alchemy, it speaks of the various transformative processes that all matter and form undergo."

However, given that no evidence is provided concerning the consensuality, or otherwise, of the crossmodal mappings chosen by Lee (2013), this approach perhaps works better as a mediation on the transformation of matter, rather than as anything particularly relevant to say about the consensual mapping of scent and sound. Importantly, Lee provides no evidence to support the claim that those combinations of musical notes that are more pleasant to listen to necessarily give rise to a perfume that is itself more pleasant to smell. Note here that simply showing that the perfume created by playing Beethoven's "Moonlight Sonata" or Mozart's "Requiem" smells good does not tell us anything about the quality, or consensuality, of the translation used. One would also want to be sure that combinations of sounds that are unpleasant deliver an unpleasant fragrance. Here, one might also consider how playing Beethoven backwards would end up creating the same perfume, while presumably having nothing like the same emotional impact. That is, this conversion of music into perfume in Lee's

[7] For a keyboard with 36 keys, every three piano keys are linked to one of 12 fragrances, whereas every four keys are linked in the case of a keyboard with 48 piano keys.

project loses any sense of temporal relationships between the elements that is such a key feature determining the meaning of music.

INSERT FIGURE 2 ABOUT HERE

2.1. Crossmodal interactions/influences of sound on scent perception

According to the results of one idiosyncratic report, some (suggestible) individuals can even be induced to believe that they have perceived a scent simply by presenting a sound. O'Mahony (1978) demonstrated this on both television and radio audiences. For instance, during a television program about the chemical senses that was aired on Granada Television in the UK (a terrestrial channel), the audience heard a tone, which they were told would cause them to experience a pleasant country smell. A number of viewers apparently wrote in to say that they had perceived the smell of grass or hay. Several people even wrote in to complain that they had suffered from attacks of hay fever and sneezing after listening to the tone! Meanwhile, several of the listeners who tuned in to a BBC Radio Bristol show reported olfactory sensations when an “ultrasonic tone” (actually silence) was played across the airwaves!

Beyond any automatic crossmodal interactions in mental imagery between sound and scent (see Spence & Deroy, 2013), various researchers have also demonstrated that changing what people hear can modify what they think about an olfactory stimulus (e.g., Velasco, Balboa, Marmolejo-Ramos, & Spence, 2014; see Spence, 2014, for a review). For example, in Velasco et al.'s study, participants rated six fragrances (specifically, the pleasant scents of lemon, orange, and bilberry, and the unpleasant scents of musk, dark chocolate, and smoked) in terms of their odour intensity, their pleasantness and their perceptual qualities (sweetness, dryness, acidity, and brightness) while listening to pleasant (consonant) music, unpleasant (dissonant) music, or broadband white noise at 70 dB over headphones. The participants sniffed one of the fragrances after having listened to a specific auditory stimulus for five seconds. However, contrary to the researchers' expectations, the congruency between the pleasantness of the music and the scent had no impact on their participants' perception of the scents. Instead, the results simply revealed that the participants rated the fragrances as smelling less pleasant, less sweet, and dryer while listening to the unpleasant white noise than when listening to either the consonant or dissonant musical stimuli.

As to why the hedonic, or affective, qualities of music and background sounds should influence the perception of olfactory stimuli, one might think only of the 'halo effect' (Deroy et al., 2013; Kenneth, 1923; Marks, 1978;), 'sensation transference' (Cheskin, 1957), or what Spence and Gallace (2011) once referred to as 'affective ventriloquism'. For instance, Kenneth talks of 'indirect associations' between smells and music resulting from *"the affect produced by smell (being) similar to the affect produced by some other stimulus"* (see Kenneth, 1923, p. 77), while Marks (1978, p. 181) suggested that sensory qualities "*talk over their common feeling*."

Seo and Hummel (2010) demonstrated that both congruent or pleasant (non-musical) sounds amplify odour pleasantness. Meanwhile, Seo, Lohse, Luckett, and Hummel (2014) reported on a series of three experiments in which they demonstrated that people match a variety of odours and background music, that congruent (to odours) background music (i.e., Christmas carols) enhanced the rated pleasantness of certain odours, and that congruent sounds also influence people's ratings of odour familiarity and identification. For example, the German participants were shown to like the smell of cinnamon more when it was presented together with (congruent) Christmas carols that when presented with sounds that were judged to be incongruent.

Zhou and Yamanaka (2018) investigated the consequences of presenting fragrances that varied in terms of their arousal potential on people's experience of music that itself was chosen to vary in terms of how arousing it was. 'Phasing', a piece of music from Steve Reich, was played at 40% vs. 80% of original tempo as the low and high arousal musical stimulus. Meanwhile, based on pre-tests, peppermint and rosemary were selected as the high alerting smells while hinoki and rose geranium were chosen as the low arousal scents. The twenty participants who took part in this study completed a total of ten conditions including each of the four scents and a no-scent baseline being paired with each of the two musical tracks. For each sound-scent combination, the participants completed a number of semantic differential scales, they rated how much they enjoyed the music, and also how much it matched to the scent. They also indicated whether they thought that the listening experience was enhanced by the scent.

Although the pattern of results was somewhat complex, there was nevertheless, some evidence to suggest that the presence of certain specific scents may have influenced the participants' emotional response to the music. For instance, the results of this Japanese study revealed that the high arousal peppermint scent may have enhanced their participant's experience of the high arousal music, while also leading to it being rated as more energetic and lively. Meanwhile, by

contrast, the presence of the low arousal hinoki scent lowered the participants' ratings of how arousing the music was relative to certain of the other conditions. The high arousal music was also evaluated as being preferable and as being more enjoyable, when the participants were in the presence of high arousal peppermint scent, rather than low arousal hinoki scent. It is, however, difficult to draw any general conclusions from these results, given that common effects depending on the arousal value of the two scents in each category (i.e., high or low) were mostly not observed. That is, the crossmodal effects that were documented appeared to be very much scent specific.

More recently, Fukumoto (2020) investigated the effect of combining two scents (chamomile-roman and peppermint, relaxing and alerting, respectively) and two pieces of music (Mars, the Bringer of War and Venus, the Bringer of Peace from Holst's The Planets) on the impressions of another group of 29 Japanese participants (see also Fukumoto, 2019; Fukumoto & Ohno, 2016). In this case, the results revealed that the four combinations of music and scent (which were each presented together for 220 seconds) were rated somewhat differently. A main effect of scent was documented on one semantic differential measure (Gentle-Furious) whereas the main effect of music was documented on three anchored semantic differential scales (Relaxing-Tension; Happy-Sad; Gentle-Furious). As such, Fukumoto argued that the crossmodal influence of scent appeared to be somewhat weaker than that of the music. Analysis of the data also revealed a significant interaction between scent and music in terms of the Good-Bad combination scale. However, contrary to Fukumoto's expectations, it was the incongruent arousal combinations that were rated more highly, perhaps suggesting some kind of crossmodal compensation effect.

Taken together, the results of the limited body of laboratory-research that has investigated the perceptual consequences of combining scent with music therefore suggest that the semantic congruency of sound and scent may have more of an influence on the latter than does hedonic congruency (Seo et al., 2014; Velasco et al., 2014; cf. Seo & Hummel, 2010). At the same time, however, the data also suggests that there may be interactions in terms of the arousal potential of scent also sometimes influencing people's response to music (Zhou & Yamanaka, 2018; see also Fukumoto, 2020).[8] Taken together, therefore, these studies demonstrate that crossmodal

---

[8] Note that the by-now extensive literature on 'sonic seasoning' (Spence, 2021b), where what people listen to has been shown to modify the taste of food and drink, falls beyond the scope of this review, despite the fact that sonic seasoning has become a major area of interest, and is closely linked to musical scents given that most of what we think we taste we actually smell (Spence, 2015b).

effects operate in both directions – that is, the addition of scent can modify people's responses to music, while what people hear can also influence what they think about what they smell. At the same time, however, the crossmodal effects appear to be quite weak and stimulus specific.

3. Scent and music combined

In this section, I want to review the various occasions in which scent and music have been combined in a public context, and the consequences, where they are known. Taking a historical stance on this question soon reveals that music and scent have, in fact, deliberately been brought together on a few occasions, though the reasons/motivation, if expressed/made clear, have tended to be quite different.

3.1. Combining scent and sound in church

Traditionally, in the West, one of the most common places where members of the general public would regularly have heard singing and music was at church. Oftentimes, incense would have been used to scent the space during (as part of) the service. As such, one might have expected there to be a semantic environmental association (cf. Seo et al., 2014) given that, for a number of centuries, these two forms of sensory stimulation would often have been experienced together. According to Heffernan and Matter (2001, p. 7), the use of incense in churches helps to mark the space out as "other". Hence, while there was no particular connection between church music and scent (incense), one might nevertheless still be tempted to say that the smell of incense can be considered congruent with choral music in that these stimuli would have co-occurred so frequently (if not necessarily because they are perceptually similar). It is, in other words, an arbitrary congruency (or correspondence), in the terminology of Lynette Walker-Andrews (1994), but the statistical correlation (or co-occurrence) between church, or choral, music and the scent of incense presumably does exist for at least a proportion of the public (cf. Classen, 1998; more so in the Catholic Church that for Methodists and Baptists). Furthermore, given Seo et al.'s (2014) results (mentioned earlier), one might expect people to rate incense as smelling more pleasant while in church, or at least while listening to church music.

3.2. Avoiding malodour

Looking back in time, it turns out that the very earliest deliberate use of scent in the context of public entertainment was to help eliminate the malodour of the masses (see Spence, 2020d, for a review). It has been suggested that this was more of a problem in the context of the cinema, given multiple daily film screenings, in contrast to the single daily performance that would have been more typical in the theatrical/operatic context. What is more, people would apparently normally get changed out of their work clothes when going to the theatre (i.e., they might don black tie, and for the men, possibly also a gardenia) in a way that they simply would not do when going to the cinema. According to the calculations of one German engineer, the cinema-goers would have had less air per person than those at the opera (Richter, 1926). In fact, during the opening decades of the 20th Century, the malodour in the cinema got so bad that deodorizing breaks, and even floating scent-dispensing blimps were sometimes used to help address the problem (again see Spence, 2020d).

Fragrant fountains were also once to be found in the lobbies of London theatres, while, fragranced program fans were occasionally featured at theatrical performances in the latter half of the 19th century (e.g., in London; Banes, 2001, p. 68; Haill, 1987; Hawking, 2015). Eugène Rimmel, the French-born, London-based perfumer, responsible for these fragrant interventions also made souvenir printed programme fans for the opening of the Gaiety Theatre, Strand, December 21st, 1868 ('Fan – Rimmel's programme fan', n.d.; Alipaz, 2015).[9] Intriguingly, the prolific perfumer also produced a Vaporizer that was advertised in the middle decades of the 19th Century (see Rimmel, 1865), as fragrancing the air at seven different entertainment houses (including Her Majesty's Theatre, St. James's Hall, Lyceum Theatre, Hanover Square Rooms, Mr. & Mrs. Howard Paul's Entertainment, Mr. Woodin's Cabinet of Curiosities), as well as on board the H. R. H. Prince of Wales royal steam yacht (see Lambert, 2013).[10]

The scented fans, fountains, and vaporizers are thought to have helped mask the malodour prior to the widespread adoption of modern hygiene practices (Jütte, 2005). And while attending a

[9] Oftentimes, the scented programs that were handed out at theatre performances were fragranced with one of Rimmel's favourite perfumes, "Royal Aquarium Bouquet" (Alipaz, 2015). Given that the scent permeated the entire program, it would presumably have acted as an effective advertisement for Rimmel and his wares. That is, there was no attempt to match the scent to the show.

[10] None of these venues were especially well known for the delivery of music events.

music concert would presumably have been more similar to attending the opera in terms of the frequency of concerts and the attire of those attending, I have been unable to find any mention of malodorous music concerts having been scented to minimize any olfactory distraction in the literature. At the same time, however, it is worth remembering that the scenting of theatres and programs was presumably a relatively rare occurrence, restricted to just a relatively small number of London venues/theatres.

More recently, the problem of malodour raised its head in the context of at least one kind of venue where music is played, specifically discos/nightclubs. The most distinctive olfactory feature of such venues following the introduction of the indoor smoking bans that came into effect some years ago was the unpleasant smell of the stale sweaty air (Schifferstein, Talke, & Oudshoorn, 2011). Intriguingly, Schifferstein and his colleagues reported how the dance club experience could be enhanced simply by introducing a pleasant scent. These researchers reported that the scents of orange, seawater, and peppermint were all equally effective, when compared to a no scent baseline, in terms of enhancing dancing activity, people's evaluation of both the music and the evening, and the latter's mood (based on almost 850 completed questionnaires). Interesting in this regard, London‘s China White nightclub started using ambient scent to improve the indoor ambience at around the same time (White, 2011). Press reports also suggested that the Luminar chain of nighclubs in the UK was pumping a rose scent through the air-conditioning to counteract the stale smell of sweat and beer (Anon. 2007).

What the research reviewed here suggests, therefore, is that while one of the first reasons for scent to be deliberately introduced into an entertainment setting was to avoid malodour, this only happened much more recently in the case of music venues such as discos/nightclubs (specifically following the smoking ban which came into effect at different times in different countries).

3.3. Scent and music in synaesthetic correspondence: Early history

In 1911, the Russian composer Alexander Nicolaevich Scriabin (1872-1915; Witztum & Lerner, 2016) premiered and published his symphonic work *Prometheus: Poem of Fire (Opus 60)* in Moscow (Baker, 2002; Gawboy & Townsend, 2012; Macdonald, 1983). Scriabin's original idea had been for his musical score to be accompanied by an optional light show (involving a part for a colour-organ), and eventually, by a simultaneous olfactory performance

as well, though, the composer failed to provide any details about the latter (Hull, 1927; Macdonald, 1983; Runciman, 1915). While the lightshow accompaniment was not performed in Scriabin's lifetime, a number of subsequent audiovisual performances of his work have taken place. One such performance was given in the Royal Albert Hall in London, on May 4th, 1972, by the London Symphony Orchestra (Griffiths, 1972). On this occasion, a spritz of Floris perfume was released into the stalls toward the end of this poorly-attended performance (MacDonald, 1983, p. 602).[11]

Scriabin's motivation is considering a scented accompaniment was derived from his interest in delivering the gesamtkunstwerk or 'total work of art'. Complicating matters somewhat though, there has been much discussion of whether or not the composer was himself a synaesthete (see Galeyev & Vanechkina, 2001; Myers, 1914) and, if so, the extent to which any indiosyncratic audiovisual synaesthetic mappings may have influenced his choice for the colour score or 'Luce'. Careful analysis of the latter, which came to light some decades after the composer's death would appear to suggest that the light element was intended to disambiguate the music. Indeed, the weight of expert opinion would appear to be against the colour concurrents being based on Scriabin's specific colour concurrents (see Gawboy & Townsend, 2012; Peacocke, 1985). It is impossible in hindsight to have any idea what Scriabin had in mind for the scent (and what the link, if any, may have been to his synaesthesia), but I think we can be pretty sure it wasn't Floris perfume (though Floris has been making perfumes in London since the 1730s).

Exactly two decades before Scriabin published his work, another multisensory performance had taken place in which music, colour, and odour were combined in a way that was deemed meaningful. In this case, the performance was built around an attempt to showcase the crossmodal correspondences, or synaesthetic mappings, between the senses. The work, *The Song of Solomon, a Symphony of Spiritual Love in Eight Mystical Devices and Three Paraphrases* (Scholes, 1970, p. 700), was performed twice in December 1891 in Paris. It was an adaptation of the Old Testament text of the *Cantique des cantiques* (Song of Songs) of Solomon (Shepherd-Barr, 1999, p. 152). It was performed in order to present a new idea of theatre as total art by engaging the audience's visual, aural, and olfactory senses. In this case, the devices specified the musical key (e.g., D major), the colour (e.g., bright orange), and the perfume (e.g., white violet) to be released by symbolist poets with handheld scent dispensers

[11] Macdonald (1983, p. 601) also notes, in passing, that *'aromas of doubtful legality'* filled the auditorium at a 1971 performance of Scriabin's work at Yale University, perhaps hinting at the fact that the crossmodal pairing may not always be the one that was intended.

(Deak, 1993, p. 155). The aim behind this work was to draw the audience's attention to the mystical correspondences connecting the senses. Unfortunately, however, the scent dispersal had not been adequately thought through (nor, more importantly, had the removal of the scents after the Symbolist poets had enthusiastically wafted them around the enclosed venue), resulting in a plethora of scents mixed together in the poorly-ventilated venue.[12] That the event was not a success hinted at by the fact that here was only ever one private (press) and one public performance of this work. The fact that the correspondences in this case may have been based, at least in part, on a synaesthete's idiosyncratic crossmodal mappings is unlikely to have facilitated the general public's enjoyment of this work either.

3.4. Semantically congruent scents

In her highly influential review of the role of scent in theatrical setting, Sally Banes (2001) laid out a number of different uses for which scent has been introduced into a live performance setting. According to Banes, the use of scent has largely been illustrative (be it of words, characters, places or actions) – in a sense, the use of scent to facilitate the storytelling. However, she also notes how scent has, on occasion, also been used to evoke a particular mood or atmosphere. Banes further points to occasions where the use of scent has served a contrastive, memorative, ritual, and/or defamiliarizing role (as summarized by Jones, 2006, p. 45). Interestingly, Banes argues that the mere pleonastic use of scent to match whatever is being shown on stage is the least interesting use of this form of scent-sory accompaniment.[13] As Banes (2001, pp. 68-69) puts it: *"... so often the use of smell seems merely iconic and illustrative, a weak link in a chain of redundancy across sensory channels that does nothing more than repeat what is already available visually and aurally."*

On occasion, semantically-meaningful scents have taken centre stage in terms of storytelling (i.e., elevating scent from a merely redundant role). On one famous occasion, in 1902, Sadakichi Hartmann attempted to transport a theatre full of people in New York across the

[12] As we will see later, much the same response was observed at an aroma jockey disco held at a Greek hotel a couple of years ago.

[13] That said, in the context of scented theme park rides, it would appear that the scents that are used are always linked semantically to the theme – such as the smell of citrus while riding on 'Soarin over the Orange groves', or the salty smell of the sea in The Pirates of the Caribbean ride (see Spence, in press-a, for a review). Interestingly, though, while it is unclear whether pleasant scents actually enhance immersion, or presence, in an experience, they will likely increase the memorability of the event.

ocean to Japan using nothing more than a selection of carefully chosen perfumes and an electric fan (Legro, 2013). In this case, the scents were meant to be congruent with / semantically match the countries that would have been visited on the journey. So, for example, meaningful scents associated with specific places, including the smell of almond for Southern France, bergamot for Italy, cedarwood for India, and the scent of carnation to represent Japan. Hartmann read out a text while a pair of geishas danced on stage. Even though Hartmann (1913) himself reports carefully choosing the scents to work when presented sequentially, commentators once again highlighted a problem with the build-up of scents in the auditorium in the single performance of this work at the New York Theatre on November 30th, 1902. Hartmann himself makes no mention of whether there was an auditory component to proceedings.

Beyond the technical challenges associated with delivering, and thereafter clearing, a large number of scents, there are also cognitive limitations with olfactory information-processing to contend with. As Avery Gilbert (2008, p. 150) notes when discussing Aldous Huxley's scent organ (mentioned earlier): "Even if the scent organ delivered odors with the brisk precision that Huxley imagined, the audience would have trouble keeping up… The human nose works on a longer time scale; it can't follow a smellody the way the ear follows a tune. Anything faster than a *largo ma non tropo* would leave an audience in the dust." It turns out that the cognitive limitations of processing olfactory information are just that much more severe in olfaction than for audition (see Gallace, Ngo, Suleitis, & Spence, 2012; Heilig, 1992; Zimmerman, 1989). Hence, given the very limited information processing ability, or bandwidth, of human olfaction the nose simply cannot hope to keep time with the rapid sequential release of scents (even if the technical solution to deliver and clear a multitude of scents in rapid succession were to be perfected). As such, given this constraint, using one scent for each movement, or perhaps a single scent for an entire piece of music would seem much more realistic that trying to match scent to specific musical notes or even musical phrases.

So, for example, here one might hypothetically consider scenting each of Vivaldi's Four Seasons with a matching scent. After all, Ranasinghe and his colleagues recently suggested releasing the scent of jasmine for spring, lemon for summer,[14] cinnamon for autumn, and a cooling mint scent to match winter as part of the two-minute multisensory VR story called Season Traveller that they developed (Ranasinghe, Jain, Tram, Koh, Tolley, Karwita, & Do,

[14] One thing to watch out for here, though, is that lemon is one of those scents that people often associate with cleaning products rather than with the citrus fruit (Holland, Hendriks, & Aarts, 2005; see Spence, 2020f).

2018). Each 30 second segment devoted to one of the seasons was accompanied by the matching scent. Given that Ranasinghe et al. demonstrated that the addition of a semantically-matching olfactory component (along with tactile effects) effectively enhanced the user's sense of presence in this VR application, one might ask why the same should not be done for a performance of Vivaldi's work.

On the other hand, sourcing the appropriate scents to accompany Holst's The Planets might well prove more difficult.[15] Simplifying matters though, one could perhaps imagine a single scent for Beethoven's 'Pastoral symphony'. At the same time, however, one would need to make sure that the introduction of a scented accompaniment is more than just a gimmick. There might also be a question of whether it is appropriate to augment the great composer's work in this way. In fact, exactly the same concerns were raised some years ago in the context of the Tate Sensorium when works of art were augmented by scent, mid-air haptics, sound, and even chocolate (see Pursey & Lomas, 2018).

It is interesting to consider whether the use of semantically-congruent scents should count as an example of the pleonastic use of scent (cf. Banes, 2001). This redundant coding has been criticised as perhaps one of the least imaginative ways in which to use scent in a theatrical/live-performance setting, and yet it has often been used (seemingly successfully) to enhance visitors' experiences on theme park rides (see Spence, in press-a, for a review). At the same time, however, the idea that one could match scent to thematic music is complicated somewhat by the fact that the music itself only indirectly represents the source theme.

One interesting example of the augmenting of a classical music concert with scents took place in 2016. The Australian Art Quartet AAQ presented an experimental project entitled "Scent of Memory" to the public in which various pieces of classical music were paired with a scented element on a related theme ('Australian Art Quartet – Scent of Memory', 2016; Sebag-Montefiore, 2016). Across two/three (the just-mentioned citations give different numbers here) sell-out shows at the Yellow House in Sydney, specifically chosen perfumes were presented while the music including work by Tchaikovsky (*String Quartet Op70/1*), Gurdjieff (*Hymns, Prayers and Rituals*), Arvo Pärt (*Fratres*), and Mountfort (*Song for Charlie*) was played. Before each piece of music was performed, international fragrance designer Carlos Huber

[15] Though note that tastes and colours have been linked to several of the planets (see Boehme, 1912, pp. 85-86; Classen, 1998, p. 167; Spence & Levitan, 2021), though not, as far as I am aware scents.

described his inspiration for the scent that was to be paired with the next piece of music, and how they were designed to evoke particular historical moments or moods. In this case, the scent delivery was very low-tech, involving the members of the audience wafting paper scent sticks under their noses while the musicians played.[16]

So, for example, while listening to Estonian Arvo Pärt's *Fratres*, the audience was taken on a journey not to the 1970s (i.e., the decade when the piece was composed), but back to a 17th century Japanese galleon on the Pacific. Huber, describes it as being loaded, with a rare cargo of spices, black pepper, Spanish leather, and frankincense. Handing out his woody, earthy perfume Nanban, with its notes of Malabar black pepper, Persian saffron, black tea, myrrh and sandalwood, Huber apparently conjured up "roasted coffee, smoky, dark" that apparently plays on Pärt's sounds (Sebag-Montefiore, 2016).[17] The scent of fresh sage, cinnamon, orange flower water, and Moroccan rosemary accompanied *Danzón No 2* by Mexican composer Arturo Márquez. The smell of cocoa represented the riches of the New World in the fragrance Anima Dulcis, paired with *Chant from a Holy Book* by George Gurdjieff. The final perfume, of Boutonnière No 7 with its faint hint of champagne and a floral flourish, was associated to Tchaikovsky's *Scherzo & Finale* from *Quartet No 1* evoking a previous era when black-tie operas were frequented by men wearing white gardenias and elegant grand dames.

3.5. Mood-enhancing scents

Both scents and music have been reported to provide an effective means of influencing our mood (see Spence, 2020f, for a review; see also FeelReal, 2021, for a multisensory headset that provides the technological solution for auditory-olfactory well-being experiences). However, one challenge in this case is the dissociation that sometimes/often occurs between the emotional tone of the music and the affective response that is induced in the listener (e.g., Cespedes-Guevara & Eerola, 2018; Juslin & Sloboda, 2010; Juslin & Västfjäll, 2008; Reybrouck & Eerola, 2017; Vuoskoski & Eerola, 2017). Given that listening to sad music may make the

[16] Given the earlier comment about the need to 'jazz-up' classical music that was mentioned at the start of this review (see Sanderson, 2018), it is interesting to see how the artistic director, James Beck, describes wanting to take classical music to a new, wider audience, saying: "We can't just sit here in black clothes" and expect concertgoers to come to us, he says. "It's about responding to those people who don't necessarily have academic or classical [music] knowledge." (quoted in Sebag-Montefiore, 2016).

[17] The latter phrase sounding like it may be describing a perceptual crossmodal correspondence between sound and scent.

listener happy, an immediate question is raised about which mood, or emtion, any scent should be matched to. One might also consider how the individual associations with scents that are too familiar can sometimes reveal very personal meanings. It should also be noted that nowadays people often appear to associate scents such as pine and lemon/citrus with cleaning products rather than their natural source objects.

4. Crossmodal correspondences between music and scent: Contemporary interest

While, as mentioned earlier, our own attempt to assess validate the consensuality of the crossmodal mappings outlined in Piesse's musical scent scale (Piesse, 1862/1891) was inconclusive, we and others have established a number of consensual crossmodal correspondences between musical attributes, such as tonal brightness (von Hornbostel, 1931), pitch (Belkin, Martin, Kemp, and Gilbert, 1997; Crisinel, Jacquier, Deroy, & Spence, 2013; Crisinel & Spence, 2012; Stevenson, Rich, & Russell, 2012), loudness (Stevenson et al., 2012), timbre (Crisinel & Spence, 2012), and vocal sounds (Macdermott, 1940; Mahdavi, Barbosa, Oliveira, & Chkoniya, 2020), types of music, and sounds of nature (Mahdavi et al., 2020), and scent.[18] For instance, Belkin and colleagues demonstrated that a range of olfactory stimuli were systematically matched with different auditory pitches. These results were later extended by Crisinel and Spence (2012), who also found that people match certain odours to the timbres of particular musical instruments (see **Figure 3A**). So, for example, the sound of the piano and woodwind instruments was found to match well with (i.e., correspond crossmodally) with fruity smells such as apricot and raspberry, while smells like musk were more commonly associated with the timbre of brass instruments instead. In terms of the auditory pitch mappings with scent, Crisinel and Spence reported that low-pitched sounds correspond with the smell of smoked, musk, dark chocolate, and cut hay, while high-pitched sounds were a better match for fruitier scents such as apple, lemon, apricot, and raspberries (see **Figure 3B**; and see Barton, 2012, for a first-person perspective from one of the participants who took part in these experiments).

INSERT FIGURE 3 ABOUT HERE

[18] Given the important role played by, and emotional connotations of music in the major vs. minor mode, it would be interesting to know what the olfactory equivalent might be? As yet, I am not aware of anyone having addressed this particular question.

Stevenson, Rich, and Russell (2012) assessed the crossmodal associations that people had with twenty different odorants. These researchers probed any crossmodal correspondences with auditory pitch (low or high) and/or with the differing loudness of sounds (i.e., quiet, medium, or loud), as well as with a range of visual, gustatory, and somatosensory attributes. So, for example, through questioning, the 18 particpants were shown to associate the smell of almond and plastic with a quiet sound, while associating lemon with a high-pitched sound. Stevenson et al. (2012, p. 614) summarize the pattern underlying their results with audition by saying: "Odours described as high-pitched tended to be easier to name and more familiar. In contrast, loudness was associated with the perceptual dimension, with 'loud' odours judged as more intense, irritating, and unpleasant." However, while individuals tended to be consistent in their crossmodal matching over time (i.e., a test-retest design was used over a two-week period), the auditory selections across particpants were not significantly different from chance, unlike for the correspondences with the other sensory modalities.

In another intriguing project that built on the crossmodal correspondences between aroma and sound, premium customers of Courvoisier cognac were sent a set of six scents that one might detect in a glass of the spirit (Crisinel, Jacquier, Deroy, & Spence, 2013). The aromas in the Nez de Courvoisier® aroma kit (Courvoisier Import Company, Deerfield, IL USA) consisted of candied orange, dried plums, roasted coffee, ginger biscuits, crème brûlée, and iris flower. Short musical excerpts (lasting for approximately 40 seconds) were developed to match each of the key aromas in the cognac. The idea was that the customer would listen to each of the musical tracks while sniffing the matching (or corresponding) aroma, thus hopefully helping to cement the link between the different instrument sounds and the aromas. Thereafter, the idea was for people to taste a glass of cognac while listening to a piece of music that incoprorated all of the sonic elements that were presented in the appropriate order to match the evolving tasting experience.

In a first experiment to assess the consensuality of this crossmodal mapping between these food aromas and musical properties, Crisinel et al. (2013) presented the six odorants from the Nez de Courvoisier® aroma kit plus an additional scent, musk (not in the kit) while assessing people's pitch, timbre, and shape associations. The results of this preliminary study revealed that higher auditory pitches were significantly matched to the odours of candied orange and iris flower while musk, roasted coffee, and ginger biscuits were matched with lower-pitched sounds instead. The crème brûlée and dried plums aromas were associated with an intermediate

pitch. The sound of the piano was matched significantly with the aroma of candied orange, dried plums, and iris flower, while musk was associated significantly with the sound of brass instruments (the other timbre choices were wind and string instruments). The other three aromas showed no significant timbral associations.

In a second study, Crisinel and her colleagues (2013) assessed how consensual the matches between scent and sound were in the laboratory. However, they found the crossmodal mappings to be only modestly successful in this case. The three musical soundtracks that had been designed by Laurent Assoulen to represent some of the aromas, partly through the use of different musical instruments (ginger cookies (strings), candied orange (harp), and crème brûlée (piano)) were tested. The results revealed that the most successful crossmodal correspondence was between the odour of candied orange and its putatively matching soundtrack. The scent of ginger biscuits was matched significantly with a track other than the one intended (namely with the crème brûlée), while the participants showed no significant preference when it came to matching the smell of crème brûlée with one of the three specially-commissioned auditory tracks. Hence, while the underlying idea of helping to structure and identify/keep track of the elements within a complex tasting experience is an intriguing one, the project highlighted some challenges. In particular, it is worth noting that while crosmodal correspondences between scent and sound were established at the featural level, one might consider mappings at the structural level (as indexed by the musical excerpts). Note here only how intramodal perceptual grouping (within audition) is much stronger than crossmodal grouping (Spence, 2015a).

Finally here, Mahdavi et al. (2020) conducted in-depth interviews with 27 people from Brazil, Portugal, and specifically covered types of music, sounds of nature, and the human voice. They had their participants suggest and elaborate on the associations envisaged. Prompts were also used whenever necessary. One example of such a prompt was “Would a perfume described in a soft voice and another described by a warm and intense voice have different scents?” The results of this cross-cultural research are summarized in **Table 1**. Taken together, the results of the research reviewed in this section, clearly highlight the existence of a number of statistically significant crossmodal correspondences between scent and sound. Such correspondences provide one basis for more artistic pairings between the senses.

INSERT TABLE 1 ABOUT HERE

As part of an ongoing collaboration between Sean Francis Conway and Brian Goeltzenleuchter a performance for scent and chamber ensemble, going by the name of 'Odophonics', took place at the San Diego Art Institute, on May 14th, 2016 (Goeltzenleuchter, 2017). The auditory element involved Minimalist structures such as consonant harmony, drones and polyrhythms to create gradual chord transformations. All the notes in this ambient soundscape can be found on Piesse's scale (see **Figure 1**). As the performers play, the corresponding scent notes were released in time. The choice of scents is derived directly from Piesse's scale. Goeltzenleuchter (2017) describes how: "Together, the musical and olfactory harmonics gradually shift. Specific to the performance is the question: What relationships exist between concurrent perceptions of smell and sound?" The performance is a jumping off point to explore Piesse's Odophone to test new propositions about how one experiences smell, particularly in relation to sound (see also Iseki & Nakamoto, 2013). However, given that, as yet, there is no empirical evidence supporting the consensuality of the crossmodal correspondences outlined in Piesse's 'Gamut of Odours', one can only imagine how much more powerful such scented musical performances might be were the mapping between scent and sound to be based on commonly-shared crossmodal associations (e.g., as documented in the work of Belkin et al., 1997; Crisinel & Spence, 2012; Mahdavi et al., 2020).

One other artist working at the interface of scent, sound, and marketing is Daniel Sonnabend (Parkin-Fairley, 2020). This composer created The Scent Constellation for the Grand Musée du Parfum, in Paris. According to Sonnabend: "The installation was an audio-visual-metaphorical representation of the process of making a perfume, with a centrepiece that looked like a perfume bottle. There were 200 perfume ingredients which made up five perfume compositions: one floral, one Chypre, a *fougère*, a Cologne and an Oriental. Each ingredient was represented by a glass prism, which had a sound associated with it. As each perfume was 'created', a laser would be beamed from the centrepiece and hit the prism for a particular ingredient, playing the sound – and the result was quite mesmerising." (quoted in Parkin-Fairley, 2020, p. 46; listen to the compositions at https://soundcloud.com/daniel-sonabend/sets/scent-constellation). Sonnabend continues: "I think the moment people start believing the correlation between scent and sound is truly possible and not just a gimmick, the use of it in e-commerce will be pushed forward. But every single project that I work on with scent breaks down that boundary in people's minds more. I don't see a difference between writing music for a film or a fragrance; you just need to be more creative. You can smell a

perfume better with your ears than you can with your eyes. Though you can still smell it best with your nose, of course!' (quoted in Parkin-Fairley, 2020, p. 47).

In terms of other commercial examples of the pairing of scent with sound, one might also consider Pantone's colour of the year in 2020, namely Classic Blue (19-4052). In this almost synaesthetic marketing campaign, Pantone worked with various creative talents to come up with what they clained to be a matching soundtrack and a matching scent. According to Fixsen (2019): *"To augment the 2020 reveal, Pantone included a twist of its own: As part of its marketing campaign, the company partnered with several brands to develop the smell, sound, taste, and texture of Classic Blue. The resulting package included a swatch of suede-like fabric from the Inside, a musk-and-sea-salt-scented candle, a blue, berry-flavored jelly, and a three-minute audio track titled "Vivid Nostalgia.""*[19] In the latter case, it is unclear to what extent the crossmodal mapping was based on synaesthesia, the crossmodal correspondences, or merely just the respective designers intuitions (see also Persolaise, 2020). Note also that no empirical evidence was provided to demonstrate that these crossmodal matches were consensual at the population level.

There have also been several attempts to create music to match a fragrance. For example, L'Orchestre Parfums fragrances (https://www.lorchestreparfum.com/en/) come complete with a QR code which, when scanned, takes you to the piece of music that was directly inspired by the scent, to listen to while smelling it (Nightingale, 2020b; see also see also https://room1015.com/content/6-music for another fragrance brand that provides music to match the scents they create). Meanwhile, some years ago, the Rafaella perfumery in Prague also ran events in which perfume and music were combined. It is worth noting that a variety of approaches to pairing have been used in such events/marketing activations, including everything from the intuitive matches of the scent/sound designer through to compositions based on specific translation of scent into sound (on occasion, based on the emerging literature on the crossmodal correspondences; there is even research emerging on the sound symbolic connection to scents; Uchida, Pathak, & Motoki, in press; see also Speed, Atkinson, Wnuk, & Majid, 2021). And see Choi, Cheok, Roman, Sugimoto, Halupka, et al. (2011) for a high-tech solution to combined scent-sound delivery.

[19] You can listen to the Audio UX track Vivid Nostalgia at https://www.youtube.com/watch?v=SVa6eQ1oRt8.

5. Scent-sory marketing

In recent decades, sensory marketers have become increasingly interested in the possibilities associated with pairing sound/music and scent in their attempts to enhance sales. Given that there has been more research on this particular combination of senses in a public setting in the context of marketing than in the context of musical performance, it is instructive to take a look at the results that have been obtained. In one intervention by Dunkin Donuts in Seoul, South Korea scent and music were combined. The smell of coffee was pumped out on a number of the city's buses when even the Dunkin Donuts jingle played on the radio (Garber, 2012). This prize-winning 'Flavour Radio' campaign united auditory and olfactory elements. Semantic congruency was also behind the Christmas-themed combination of scent and sound in Spangenberg, Grohmann, and Sprott's (2005) marketing study. The latter researchers assessed the impact on consumers of presenting seasonal Christmas music (vs. non-Christmas music), seasonal scent (vs. no scent), or combining the two sensory cues in either a congruent or incongruent manner in perception of a mock store set-up (cf. Seo et al., 2014). The results showed that people's evaluation of the store was enhanced specifically by the presence of congruent scent and sound combination. By contrast, it was the congruency (or matching) of the arousal potential of auditory and olfactory stimuli was of more interest to Mattila and Wirtz (2001), who combined no, low, and high arousal scent with no, low, and high arousal music at a retail outlet. Their results demonstrated that when the ambient scent and music were congruent in terms of their arousing qualities, the consumers (N = 270, mostly women) rated the environment significantly more positively, exhibited higher levels of approach and impulse buying behaviours, and experience enhanced satisfaction than when these environmental cues (i.e., relaxing vs. energizing) conflicted with each other). Moreover, only when scent and music was combined was there an effect on impulse purchasing.

However, it is important to stress that a positive effect of combining scent and sound have not always been observed in a marketing/multisensory design context. So, for example, Morrin and Chebat (2005) assessed the impact of scent, music or both on impulse purchases by shoppers at a North American mall (N = 774 shoppers). Intriguingly, however, while the presentation of music led to a significant increase in sales, further adding scent actually led to a decrease in sales, compared to baseline. (Adding scent but not sound resulted in no change in impulse purchases.) It is difficult to know in hindsight whether the decline in sales in the multisensory

condition may have reflected an example of sensory overload (Malhotra, 1984) or sensory incongruence. Finally here, Fenko and Loock (2014) investigated the effects of combining lavender scent, instrumental background music, or both on patients' (N = 117) anxiety in a plastic surgeon's waiting room (cf. Becker, Chambliss, Marsh, & Montemayor, 1995). While presenting either of the senses individually was shown to lead to a significant reduction in anxiety, here was no benefit from combining the senses.

Thus, while sensory marketing/multisensory design studies demonstrate that combining the senses can significantly influence people's perception and behaviour (Garber, 2012; Mattila & Wirtz, 2001; Spangenberg et al., 2005), there have also been situations in which combining the senses has either had no impact (Fenko & Loock, 2014), or else even a negative effect on sales (Morrin & Chebat, 2005). By contrast to the marketing/multisensory design literature, it is noticeable how there is currently no empirical evidence that the audiences at any of the scented music events described in the previous sections actually benefited from a significantly enhanced multisensory experience. In other words, while the laboratory research suggests that adding the appropriate scent can enhance the audience's response to music, and while occasional first person reports have enthused about the experience of conjoining these senses,[20] there have been no adequately powered empirical studies, excepting Schifferstein et al.'s (2011) study where fragrances were added to a disco/nightclub in order to mask the ambient malodour following the smoking ban. And staying with the scented disco theme, aroma jockeys have also been pairing music with scent for decades now.

6. Scent-sory disco: Welcome to the aroma jockey

Aroma jockeys, such as Austrian artist Erich Berghammer "ODO7" (OdO7, 2010; http://odo7.com/), curate popular music events in which a sequence of aromas are delivered via wind devices (often Dyson bladeless fans) while the music is blasting out (Chesters, 2017; Orb Mag, 2018).[21] However, beyond the novelty of such a sensory combination, there does not

[20] Such as the following from James Beck, the artistic director of the AAQ, who claims that "Scent and music together are an intoxicating combination, potent and overpowering." Or the following from The Scented Letter, (2020, p. 3): "Persolaise reveals his perfume 'playlist'. As he puts it, 'Making the effort actively to listen to a whole piece of music is just as rich and memorable an experience as plunging into the complexity of a perfume. But what's even more powerful is when you combine the two.'"

[21] However, while the experience was certainly novel, the first author's experience of one such event left him wondering about the problems of getting the intensity and sequencing right. There was a heady onslaught of scent

appear to be much sign that such multisensory dance events have caught on in the mainstream since aroma jockeys such as Berghammer started practicing their scented music events a couple of decades ago, though scented DJ-ing is apparently popular amongst the deaf (Chesters, 2017). From the press descriptions of such events, and the interviews with practitioners such as Berghammer, it would appear that they use various different approaches when deciding which scent to pair with a particular track. According to Berghammer: "In order to interpret popular songs with scent, you need to be able to listen to a song with your nose, meaning you naturally choose scents that harmonise with what you're hearing." (quoted in Chesters, 2017).[22]

It is interesting to consider the performance of the aroma jockey in the context of classical music concert, as opposed to when DJ-ing. Berghammer apparently once performed alongside the Amsterdam chamber orchestra, describing how he used: "classical scents like cedar wood and neroli [bitter-orange-blossom oil]." (Chesters, 2017). Here the crossmodal mapping would appear to be semantically based. Note here also how the program of a classic music concert tends to be fixed far in advance, while the choice of music in a DJ set often feels to be improvised on the fly. As such, expressing the immediate/spontaneous responses to music would appear to make more sense in the latter context than the former.

Elsewhere, Berghammer's description of his choice of scented accompaniment for music when DJ-ing would appears to reference everything from perceptual similarity ("R&B or rap sounds very much like spices, floral scents and spearmint.") through to synaesthesia ("There are certain rules for creating synaesthesia and harmony with scent – creating "smound", a perception or sense experience produced from the convergence of scents and sounds in the brain."),[23] matching the arousal properties of music (" Energetic house smells like grapefruit blended with castoreum essence.") through scent, through to modulating mood/arousal ("It can be unbelievably pleasant to smell strong menthol deep into the night when dancing in a techno club."; all quotes from Chesters, 2017). While Berghammer aka oDo7 is perhaps the most

– hinting (once again), at the difficulty of scenting large spaces, and, more important, clearing out the scents to avoid a build-up.

[22] In the scented disco I attended in Greece it appeared to be more spontaneous feeling than anything more systematic. Furthermore, and perhaps obviously, it felt like the scents were chosen in response to the music that was playing, rather than vice versa.

[23] For those who were wondering, 'smound' is a term that first appeared in an article by Peeples (2010) in *Scientific American*. The term emerged from research in the animal model demonstrating olfactory-auditory sensory convergence in the olfactory tubercle (Wesson & Wilson, 2010, 2011; see also Cohen, Rothschild, & Mizrahi, 2011; Zhou, Lane, Noto, Arabkheradmand, Gottfried, Schuele, et al., 2019).

famous aroma jockey, he is by no means the only one, with a number of other aroma jockeys, going by names such as Dr. Perfume in Mexico, and Aroma Jockey Jerome, also offering their services online around the world. Nevertheless, despite their growing number this is undoubtedly still a niche offering.

7. Combining scent and sound in the absence of sight

Given the ubiquitous phenomenon of visual dominance, one might imagine that scented music experiences might be more successful in the absence of sight (i.e., when vision is removed). Indeed, in recent years, some pop concerts have occasionally been help in the dark (including performances of John Metcalfe's 'A Darker Sunset'), perhaps inspired by the success of dine-in-the-dark restaurants (Spence & Piqueras-Fiszman, 2012, 2014). Powerful examples of storytelling in the dark that incorporated scents and non-musical auditory stimuli have also taken place (Delaunay, 2014).

One much-publicized scented music performance that took place in near darkness at The Guggenheim Museum in New York was *Green Aria: A Scent Opera*. This was *'described as a beguiling 30-minute work'* with scents (30 distinctively-named fragrances) created by the French perfumer Christophe Laudamiel (Alter, 2009; Khamsi, 2009; Lubow, 2009; Tommasini, 2009). In this case, a high-tech solution to scent delivery was chosen with every seat wired up with its own 'scent microphone' – a tube that audience could bring as close to their nose as they liked. At the start of this scented musical experience, five elements and 18 supporting characters were introduced. As each character was announced, the audience heard the music and smelled the scent associated with the character. For example, *"Fire + Smoke had crinkling electronic sounds and a piercing, burnt-ash scent."* The audience were seated in near darkness, experiencing an abstract drama of sound and scent. The show was premiered on May 31st and June 1st. Khamsi (2009, p. 513) notes that: "The unconventional music of the opera — written by composers Nico Muhly and Valgeir Sigurðsson — does not contain words but consists instead of sung sounds, notes played by orchestra instruments and electronic elements. When the music calls for a chord of voices, the scent speakers, too, release a 'chord' of corresponding odours." The latter would presumably have made Piesse happy.

Along similar lines to the *Green Aria,* D'Errico (2018) describes an audio-olfactory production of *Les Parfums de l'Âme* by French playwright Violaine de Carné in a theatre that was suffused

with incense while listening to a requiem. A further 12 odours were diffused, each being linked to a specific character, or to one of their memories. Questionnaires completed by more than 300 of those who had attended one of the performances (as well as 35 individual interviews) indicated that the majority of people (77%) were satisfied with the olfactory element of proceedings – both the odour experience itself and the odour release system (Salesse & Domisseck, 2015). Unfortunately, however, there was no direct comparison of scented to unscented version of the experience to really help quantify how much better the musical experience was with scent. Thus, when combined with the various events described earlier, it turns out that scented auditory experiences (either with or without a visual element) are not entirely unheard of, although they are pretty rare, and what is also noticeable is how such scented events never seem to last for more than two or three performances at most. The one exception to the latter claim being the scented disco events put on by aroma jockeys such as Austrian artist Erich Berghammer "ODO7". Having taken a look at the combination of scent and sound in the public sphere, it is time finally to briefly consider the possible future combination of these two senses in the home environment.

8. Scenting sonic experiences in the home environment

Having taken a look at the pairing (or matching) of scents and sounds in the public sphere it is now time to consider how such experiences might one day be brought into the private (or home) sphere. One of the challenges when it comes to the home environment concerns the challenging problem of scent delivery. Interestingly, while a number of digitally-controlled scent solutions have been proposed in recent years (see Spence, in press-a), the number of scents that the majority of such devices can provide is likely to be too limited to provide sufficient range to accompany a diverse musical performance. Most devices offer little more than a handful of odorants. The important point to stress here is that it is simply not possible to create scents by mixing a given number of base odorants in the same way in which colour printers, say, are capable of creating any colour simply by mixing together red, yellow, blue, and black (see Sebag-Montefiore, 2015; Spence, Ranasinghe, Velasco, & Obrist, 2017).[24] Should the aim of

[24] This issue was highlighted by Luca Turin in a newspaper article: *"Turin believes that Smell-O-Vision has never taken off because, unlike colour TV, smell has no primaries that can be mixed to make endless combinations. "You cannot create an enormous palate of smells the way you can with [just three primary] colours," he explains. "And that is a fundamental technological problem."* (quoted in Sebag-Montefiore, 2015).

adding scent to a musical experience be simply to enhance the mood/emotion evoked by the music (Sammler, Grigutsch, Fritz, & Koelsch, 2007), then a limited number of odorants are presumably needed (Spence, 2020f, for a review). However, it is unlikely that delivering a sequence of 25-25 scents, as often considered in the early days of scented cinema is likely to be possible/successful (see Spence, 2020d, for a review).

In some of the latest scent-enabled tech (such as vaqso, see Cakebread, 2017; FeelReal, 2021), can fit onto standard VR headsets (such as Occulus Rift; or Magic Leap). Given that these solutions work with standard VR kit, this would open up the very real possibility of recreating Scriabin's Prometheus (if only we knew what scent the Russian composer had in mind). More interesting to your present author might be the delivery of the 'Last five minutes' experience (see Delauney, 2014). However, the existence of the 'fundamental misattribution error' (Spence et al., 2017) should not be neglected. This is the name given to the fact that as visually-dominant creatures, we tend to attribute our pleasures to what we see (Hutmacher, 2019), or possibly to what we hear (in the context of a music concert), rather than to wat we smell. As such, consumers may be unlikely to buy the scent refills that will be needed to bring scented entertainment into the home, no matter whether one is talking about scented home music or scented television.

Sensory apps have come onto the market in the last couple of years that allow the user to take a picture of a wine bottle label in order to access recommendations for the matching music (http://winelistening.com/). Why not do the same for bottles of perfume, one might ask? After all, there is is more published research matching fragrance components to their auditory properties than there is matching wine-specific chemosensory attributes to sound (e.g., see Music & Scent, 2020).[25]

9. Conclusions

[25] One might also wonder whether knowing about a person's musical preferences might provide a better clue to their fragrance likes that assessing their preferred colour, as is purported done at fragrance counters in Germany, for instance (Spence, 2002, 2020g).

As this review of the literature has hopefully made clear, scented musical events, although rare, are not entirely unheard of. However, it should be said that the majority of them have not been considered especially successful, often due to problems with scent delivery or clearance (Hartmann, 1913; Legro, 2013; Shepherd-Barr, 1999). However, even on those occasions where the problems with scent delivery have been resolved, be it with a high-tech Alter, 2009; Delaunay, 2014; Khamsi, 2009; Lubow, 2009; Tommasini, 2009) or low-tech solution (Sebag-Montefiore, 2016), it is noticeable how limited the number of such events are (typically never extending beyond just one to three events). Scented musical public events would, then, seem to be more of a niche undertaking than anything else. As such, it would seem unlikely that they will be extended into the home any time soon, despite the emergence of various sensory apps, and despite the growing interest from those sensory marketers wanting to know whether scent can be meaningfully conveyed by sound (Mahdavi et al., 2020; Parkin-Fairley, 2020; cf. Crisinel et al., 2013).

What is also striking from the literature reviewed here is that the marketers'/multisensory designers' attempts to combine scent and sonic (music) elements have only occasionally been successful (Garber, 2012; Mattila & Wirtz, 2001; Spangenberg et al., 2001), and have often been disappointing (Fenko & Loock, 2014; Morrin & Chebat, 2005). It is especially important to consider such mixed results in the context of the absence of empirical research assessing the effects of adding a scented element to public music events (excepting Schifferstein et al.'s, 2011, study of scents to mask the malodour in the disco).

Those who have deliberately chosen to combine scent with sound in a public music setting have done so for a number of different reasons. They have chosen to pair the senses based on masking malodour (Rimmell, 1865; Schifferstein et al., 2011), semantics, affective valence and mood induction, synaesthesia and crossmodal correspondences. The introduction of semantically meaningful scents has been used to augment what is heard, and/or to help tell stories (Delaunay, 2014; Hartmann, 1913; Legro, 2013). However, it is important to note that in going beyond what the composer intended, questions are raised (assuming that is, the scent is effective; Pursey & Lomas, 2018). On occasion, scent and music have been paired to help illustrate the correspondences, or perceptual similarity between scent and sound (e.g., Fixsen, 2019; Goeltzenleuchter, 2017; Sebag-Montefiore, 2016; Shepherd-Barr, 1999). However, in the majority of such cases, the crossmodal mapping has been based on Piesse's untested correspondences found it his Gamut of Odours (see **Figure 1**) rather than on the scientifically-

validated crossmodal correspondences that have been established by researchers over the last quarter of a century or so.

In rare cases, the scents are used to move beyond (or complement) the music (Sebag-Montefiore, 2016), and/or, to set, signal, induce and/or help maintain a specific mood (Chesters, 2017; Feelreal, 2021). Bane (2001) has provided a helpful list of the various uses to which scents have been creatively put in the context of live performance (specifically theatre), and a number of her categories are undoubtedly relevant when it comes to thinking about the deliberate combination of sound and scent, be it is a live or digital context. A number of those working in this space have wanted to probe the nature of the relationship between simultaneous scent and sound, by asking, for example, whether it is possible to experience cross-sensory harmony (Chesters, 2017). As aroma jockey Erich Berghammer put it in one interview "I recall themoment when two friends and I smelled the scents with music in perfect harmony for the very first time, and then I knew that scent would be my future medium." (quoted in Chesters, 2017). However, it is important to recognize that intramodal perceptual grouping is typically stronger than crossmodal grouping (see Spence, 2015a), and this may cause problems when going from the crossmodal correspondences that have typically been established at the featural level to the musical compositions that scent is typically paired with in real-world multisensory events. At the same time, however, technical problems with scent delivery/clearance (Spence, 2020d; Spence, 2021a), as well as fundamental differences in information processing between the senses of sound and smell (Gallace et al., 2012; Zimmrman, 1989), make such crossmodal synergies much more difficult to work with (Gilbert, 2008; Goeltzenleuchter, 2017), despite the fact that these two senses are much more evenly matched than when either sense is compared/combined with the dominant visual sense (see Deroy et al., 2013; Hutmacher, 2019). The presence of the 'fundamental misattribution error' (Spence et al., 2017), and the difficulties associated with the digital delivery of scent are also highlighted as factors that are likely to limit the uptake of scented musical entertainment in the home. As such, it would seem most likely that the crossmodal mapping in the home will likely to in the opposite direction with the emergence of sensory apps that can convert fragrance (e.g., perfume bottle labels) into sound and music.

Acknowledgments

This research was supported by the Rethinking the Senses grant from the AHRC(UK) awarded to Charles Spence (AH/L007053/1).

REFERENCES

Ackerman, D. (2000). *A natural history of the senses*. London, UK: Phoenix.

Alipaz, L. E. (2015). There's nose business like show business: Fragrance in the performing arts, Part I. *Fragrantica*. https://www.fragrantica.com/news/There-s-Nose-Business-Like-Show-Business-Fragrance-in-the-Performing-Arts-Part-I-7363.html.

Alter, A. (2009). The opera hasn't even opened, but we know it smells. *The Wall Street Journal,* **May 20th**. https://www.wsj.com/articles/SB124277733367437141.

Anon. (2007). Luminar to fight smoking ban with sex toys and scent. *Property Week*, **May 18th**. https://www.propertyweek.com/news/sex-toys-and-scent-to-replace-smoke-in-luminar-clubs-/3087374.article.

Athanasopoulos, G., Eerola, T., Lahdelma, I., & Kaliakatsos-Papakostas, M. (2021). Harmonic organisation conveys both universal and culture-specific cues for emotional expression in music. *PLoS ONE*, **16(1)**:e0244964. https://doi.org/10.1371/journal.pone.0244964.

Australian Art Quartet – Scent of Memory, 2016; https://yellowhousesydney.com.au/workshops/2016/10/13/the-scent-of-memory.

Baker, J. (2002). *Prometheus* and the quest for color-music: The world premiere of Scriabin's *Poem of Fire* with lights, New York, March 20, 1915. In J. Leggio (Ed.), *Music and modern art* (pp. 61-95). New York, NY: Routledge.

Banes, S. (2001). Olfactory performances. *TDR/The Drama Review*, **45(1)**, 68-76.

Barton, C. (2012). How sound and smell can create perfect harmony. *The Guardian*, **October 22nd**. https://www.theguardian.com/science/2012/oct/22/sound-and-smell-create-harmony.

Baus, O., & Bouchard, S. (2017). Exposure to an unpleasant odour increases the sense of presence in virtual reality. *Virtual Reality*, **21**, 59-74.

Becker, N., Chambliss, C., Marsh, C., & Montemayor, R. (1995). Effects of mellow and frenetic music and stimulating and relaxing scents on walking by seniors. *Perceptual and Motor Skills*, **80**, 411-415.

Belkin, K., Martin, R., Kemp, S. E., & Gilbert, A. N. (1997). Auditory pitch as a perceptual analogue to odor quality. *Psychological Science*, **8**, 340-342.

Boehme, J. (1912). *The signature of all things*. https://www.sacred-texts.com/eso/sat/index.htm.

Bull, M. (2000). *Sounding out the city: Personal stereos and the management of everyday life*. Oxford, UK: Berg.

Bull, M., & Black, L. (Eds.). (2005). *The auditory culture reader*. Oxford, UK: Berg.

Caivano, J. L. (1994). Color and sound: Physical and psychophysical relations. *Color Research and Application*, **19(2)**, 126-133.

Cakebread, C. (2017). Virtual reality gets smelly thanks to this Japanese startup. *Business Insider*, **January 27th**. https://www.businessinsider.com/japanese-startup-vaqso-adding-smells-virtual-reality-2017-6.

Carù, A., & Cova, B. (2005). The impact of service elements on the artistic experience: The case of classical music concerts. *International Journal of Arts Management*, **7**, 39-54.

Cespedes-Guevara, J., & Eerola, T. (2018). Music communicates affects, not basic emotions—A constructionist account of attribution of emotional meanings to music. *Frontiers in Psychology*, **9**:215. doi: 10.3389/fpsyg.2018.00215.

Cheskin, L. (1957). *How to predict what people will buy*. New York, NY: Liveright.

Chester, N. (2017). I spoke to an 'aroma jockey', who DJs smells for deaf audiences. *Vice*, **March 15th**. https://www.vice.com/en/article/9amnq7/i-spoke-to-an-aroma-jockey-who-djs-smells-for-deaf-audiences.

Choi, Y., Cheok, A. D., Roman, X., Sugimoto, K., Halupka, V., et al. (2011). Sound perfume: Designing a wearable sound and fragrance media for face-to-face interpersonal interaction. In *Proceedings of the 8th International Conference on Advances in Computer Entertainment Technology*, 4. ACM Press.

Classen, C. (1998). *The color of angels: Cosmology, gender and the aesthetic imagination*. London, UK: Routledge.

Classen, C., Howes, D., & Synnott, A. (1994). *Aroma: The cultural history of smell*. London, UK: Routledge.

Cohen, L., Rothschild, G., & Mizrahi, A. (2011). Multisensory integration of natural odors and sounds in the auditory cortex. *Neuron*, **72**, 357-369. doi: 10.1016/j.neuron.2011.08.019

Cooke, E., & Myin, E. (2011). Is trilled smell possible? How the structure of olfaction determines the phenomenology of smell. *Journal of Consciousness Studies*, **18(11-12)**, 59-95.

Crisinel, A.-S., Jacquier, C., Deroy, O., & Spence, C. (2013). Composing with cross-modal correspondences: Music and smells in concert. *Chemosensory Perception*, **6**, 45-52. 10.1007/s12078-012-9138-4

Crisinel, A.-S., & Spence, C. (2012). A fruity note: Crossmodal associations between odors and musical notes. *Chemical Senses*, **37**, 151-158. doi:10.1093/chemse/bjr085.

Deak, F. (1993). *Symbolist theater: The formation of an avant-garde*. Baltimore: Johns Hopkins University Press.

Delaunay, N. (2014). Dutch scientists recreate deaths of JFK, Diana, Whitney Houston through smell. *The Sydney Morning Herald*, **December 26th**. https://www.smh.com.au/technology/dutch-scientists-recreate-deaths-of-jfk-diana-whitney-houston-through-smell-20141226-12e6uw.html.

D'Errico, A. (2018). The nose onstage: Olfactory perceptions and theatrical dimension. In V. Henshaw, K. McLean, D. Medway, C. Perkins, & G. Warnaby (Eds.), *Designing with smell: Practices, techniques and challenges* (pp. 227-236). New York, NY: Routledge.

Deroy, O., Crisinel, A.-S., & Spence, C. (2013). Crossmodal correspondences between odors and contingent features: Odors, musical notes, and geometrical shapes. *Psychonomic Bulletin & Review*, **20**, 878-896. doi 10.3758/s13423-013-0397-0.

Deroy, O., & Spence, C. (2013). Why we are not all synesthetes (not even weakly so). *Psychonomic Bulletin & Review*, **20**, 643-664.

Di Stefano, N., Murari, M., & Spence, C. (2021). Crossmodal correspondences in art and science: Odours, poetry, and music. In N. Di Stefano & M. T. Russo (Eds.), *Olfaction: An interdisciplinary perspective*. Springer.

Edwards, M. (1984). *The fragrance manual* (1st Ed.). La Quinta, CA: Crescent House Publishing.

Fakotakis, N. D. (2018). Synesthesia: The smell of musical notes. *Evolving Science*, **June 13th**. https://www.evolving-science.com/information-communication/synesthesia-smell-musical-notes-00688.

Feelreal (2021). Feelreal sensory mask. Retrieved from bit.ly/2Vg1fhS (last accessed May 20th, 2021).

Fenko, A., & Loock, C. (2014). The influence of ambient scent and music on patients' anxiety in a waiting room of a plastic surgeon. *HERD: Health Environments Research and Design Journal*, **7(3)**, 38-59.

Finn, A. (2008). Can you smell classical music? *The Guardian*, **December 8th**. https://www.theguardian.com/stage/theatreblog/2008/dec/08/perfume-robert-winston-music. (Accessed July 9, 2021)

Fixsen, A. (2019). The 2020 Pantone color of the year is Classic Blue. *Architectural Digest* **December 5th**. https://www.architecturaldigest.com/story/pantone-color-of-the-year-2020#:~:text=To%20ring%20in%20yet%20another,%2C%2019%2D4052%20Classic%20Blue.

Fukumoto, M. (2020). Investigation of main effect of scent in cross-modal association between music and scent. *International Journal of Affective Engineering*. Doi: 10.5057/ijae.IJAE-D-20-00006.

Fukumoto, M. (2019). Investigation of music and fragrance from perspective of cross-modal effects. *Proceedings of the 20th International Symposium on Advanced Intelligent Systems and 2019 International Conference on Biometrics and Kansei Engineering*, 16-20.

Fukumoto, M., & Ohno, Y. (2016). A fundamental investigation of cross-modal effects of music and scent on relaxation feelings. *Proceedings of the 2nd International Symposium on Affective Science & Engineering*, A1-1.

Gallace, A., Ngo, M. K., Sulaitis, J., & Spence, C. (2012). Multisensory presence in virtual reality: Possibilities & limitations. In G. Ghinea, F. Andres, & S. Gulliver (Eds.), *Multiple sensorial media advances and applications: New developments in MulSeMedia* (pp. 1-38). Hershey, PA: IGI Global.

Garber, M. (2012). The future of advertising (will be squirted into your nostrils as you sit on a bus). *The Atlantic*, **July 26th**. https://www.theatlantic.com/technology/archive/2012/07/the-future-of-advertising-will-be-squirted-into-your-nostrils-as-you-sit-on-a-bus/260283/.

Gawboy, A. M., & Townsend, J. (2012). Scriabin and the possible. *MTO*, **18(2)**, 1-21.

Gilbert, A. (2008). *What the nose knows: The science of scent in everyday life*. New York, NY: Crown.

Goeltzenleuchter, B. (2017). Odophonics: A performance for scent and chamber ensemble. https://www.bgprojects.com/home/2017/9/4/odophonics.

Griffiths, P. (1972). Stravinsky, Skryabin. *The Musical Times*, **113,** 682.

Haill, C. (1987). 'Buy a bill of the play!' *Apollo*, **126 (New Series 302)**, 284.

Hartmann, S. (1913). In perfume land. *Forum (1886-1930) (NY)*, **50(August)**, 217-228.

Hawking, F. K. (2015). The (under)use of scent in theatre. *Isis*, **February 22nd**. http://isismagazine.org.uk/2015/02/the-underuse-of-scent-in-theatre/.

Heffernan T. J., & Matter, E. A. (Eds.). (2001). *The Liturgy of the Medieval Church*. Kalamazoo, MI: Medieval Institute Publication.

Heilig, M. L. (1992). El cine del futuro: The cinema of the future. *Presence: Teleoperators, and Virtual Environments*, **1**, 279-294. (Reprinted from *Espacios*, **23–24**, 1955).

Henshaw, V., McLean, K., Medway, D., Perkins, C., & Warnaby, G. (Eds.). (2018). *Designing with smell: Practices, techniques and challenges*. New York, NY: Routledge.

Holland, R. W., Hendriks, M., & Aarts, H. (2005). Smells like clean spirit. Nonconscious effects of scent on cognition and behavior. *Psychological Science*, **16**, 689-693.

Hutmacher, F. (2019). Why is there so much more research on vision than on any other sensory modality? *Frontiers in Psychology*, **10**:2246. doi: 10.3389/fpsyg.2019.02246.

Huxley, A. (1932). *Brave new world*. New York, NY: Harper & Row.

Ischer, M., Baron, N., Mermoud, C., Cayeux, I., Porcherot, C., Sander, D., & Delplanque, S. (2014). How incorporation of scents could enhance immersive virtual experiences. *Frontiers in Psychology*, **5**:736.

Iseki, M., & Nakamoto, T. (2013). Interactive art of sound and scent using an olfactory display. *Proceedings of the Interaction*, **2013**, 570-574 (in Japanese).

Jones, C. A. (Ed.). (2006). *Sensorium: Embodied experience, technology, and contemporary art*. Cambridge, MA: MIT Press.

Juslin, P. N., & Sloboda, J. A. (2010). *Handbook of music and emotion: Theory, research, applications*. Oxford, UK: Oxford University Press.

Juslin, P. N., & Västfjäll, D. (2008). Emotional responses to music: The need to consider underlying mechanisms. *Behavioral and Brain Sciences*, **31**, 559-575.

Jütte, R. (2005). *A history of the senses: From antiquity to cyberspace* (trans. James Lynn). Cambridge, UK: Polity Press.

Kenneth, J. H. (1923). Mental reactions to smell stimuli. *Psychological Review*, **30**, 77-79.

Kettler, A. (2015). Making the synthetic epic. *The Senses and Society*. **10(1)**, 5-25. doi:10.2752/174589315X14161614601682.

Khamsi, R. (2009). Smells like green spirit. *Nature*, **459**, 513.

Lam, C.-W. (1994). *The lost tradition: Changing interpretations of music in the three Chinese Confucian ritual classics from the Han to the Qing dynasty*. Durham theses, Durham University. Available at Durham E-Theses Online: http://etheses.dur.ac.uk/1023/.

Lambert, J. A. (2013). Rimmel's scented world. *Blog*, **February 1st**. http://blogs.bodleian.ox.ac.uk/jjcoll/2013/02/01/rimmels-scented-world/.

Lee, C. H. (2013). *Essence in space*. https://www.changheelee.com/essence-in-space.html.

Legro, M. (2013). A trip to Japan in sixteen minutes. *Believer*, **98(May)**. http://www.believermag.com/issues/201305/?read=article_legro.

Lubow, A. (2009). The smell of music. *New York Art*, **June 1st**. http://nymag.com/arts/art/features/56912/.

Macdonald, H. (1983). Lighting the fire: Skryabin and colour. *The Musical Times*, **124**, 600-602.

Mahdavi, M., Barbosa, B., Oliveira, Z., & Chkoniya, V. (2020). Sounds of scents: Olfactory-auditory correspondences in the online purchase experience of perfume. *Revista Brasileira de Gestao de Negocios*, **22(4)**, 836-853.

Malhotra, N. K. (1984). Information and sensory overload. Information and sensory overload in psychology and marketing. *Psychology & Marketing*, **1(3-4)**, 9-21.

Marks, L. E. (1978). *The unity of the senses: Interrelations among the modalities*. New York, NY: Academic Press.

Marks, P. (1999). An Irish classic given cartoon form. *The New York Times*, **October 8th**, E3.

Mattila, A. S., & Wirtz, J. (2001). Congruency of scent and music as a driver of in-store evaluations and behavior. *Journal of Retailing*, **77**, 273-289.

Maycock, R. (1989). Truly taking the pith (Review). *The Independent*, **December 9th**, 33.

Macdermott, M. M. (1940). *Vowel sounds in poetry: Their music and tone colour*. London, UK: Kegan Paul.

McGann, J. P. (2017). Poor human olfaction is a 19th-century myth. *Science*, **356**, eaam7263.

McGinley, M., & McGinley, C. (2018). Olfactory design elements in theatre: The practical considerations. In V. Henshaw, K. McLean, D. Medway, C. Perkins, & G. Warnaby (Eds.), *Designing with smell: Practices, techniques and challenges* (pp. 219-226). New York, NY: Routledge.

Morrin, M., & Chebat, J. C. (2005). Person-place congruency: The interactive effects of shopper style and atmospherics on consumer expenditures. *Journal of Service Research*, **8**, 181-191.

Music & Scent (2020). *The Scented Letter*, **42 (whole issue)**.

Myers, C. S. (1914). Two cases of synaesthesia. *British Journal of Psychology*, **7**, 112-117.

Newton, I. (1952). *Opticks or a treatise on the reflections, refractions, inflections & colours of light*. New York, NY: Dover Publications. (Original work published 1730).

Nightingale, S. (2020a). Note perfect. *The Scented Letter*, **42**, 11-16.

Nightingale, S. (2020b). A scented symphony. *The Scented Letter*, **42**, 18-21.

North, A., & Hargreaves, D. (2008). *The social and applied psychology of music*. Oxford, UK: Oxford University Press.

O'Mahony, M. (1978). Smell illusions and suggestion: Reports of smells contingent on tones played on television and radio. *Chemical Senses and Flavour*, **3**, 183-189.

Orb Mag (2018). Erich Berghammer reinvents the perception of scent as aroma jockey ODO7. *Orb Mag*, **April 18th**. https://www.orbmag.com/features/erich-berghammer-reinvents-the-perception-of-scent-as-aroma-jockey-odo7/

Parkin-Fairley, C. (2020). So, what does cis-3-hen-1-ol sound like? *The Scented Letter*, **42**, 45-47.

Parr, D. R. (2018). Indeterminate ecologies of scent. In V. Henshaw, K. McLean, D. Medway, C. Perkins, & G. Warnaby (Eds.), *Designing with smell: Practices, techniques and challenges* (pp. 259-269). New York, NY: Routledge.

Peacock, K. (1985). Synesthetic perception: Alexander Scriabin's color hearing. *Music Perception*, **2**, 483-506.

Peeples, L. (2010). Making scents of sounds. *Scientific American*, **302(4),** 28-29.

Perfumery Organ (2015/17). Perfumery Organ Project. Intercommunication Centre (ICC). https://www.ntticc.or.jp/en/archive/works/perfumery-organ/.

Persolaise, S. (2020). Listening to scent. *The Scented Letter*, **42**, 28-31.

Piesse, C. H. (1862/1891). *Piesse's art of perfumery* (5th Ed.). London, UK: Piesse and Lubin. Downloaded from http://www.gutenberg.org/files/16378/16378-h/16378-h.htm.

Piesse, G. W. S. (1867). *The Art of Perfumery and the Methods of Obtaining the Odors of Plants: With Instructions for the Manufacture of Perfumes for the Handkerchief, Scented Powders, Odorous Vinegars, Dentifrices, Pomatums, Cosmetics, Perfumed Soap, Etc., to which is Added an Appendix on Preparing Artificial Fruit-essences, Etc*. Philadelphia: Lindsay & Blakiston.

Pridmore, R. W. (1992). Music and color: Relations in the psychophysical perspective. *Color Research & Application*, **17**, 57-61.

Pursey, T., & Lomas, D. (2018). Tate Sensorium: An experiment in multisensory immersive design. *The Senses and Society*, **13(3)**, 354-366.

Ranasinghe, N., Jain, P., Tram, N. T. N., Koh, K. C. R., Tolley, D., Karwita, S., & Do, E. Y.-L. (2018). Season traveller: Multisensory narration for enhancing the virtual reality experience. In R. Mandryk & M. Hancock (Eds.), *Proceedings of the 2018 CHI conference on human factors in computing systems* (pp. 1-13). New York, NY: ACM.

Reybrouck, M., & Eerola, T. (2017). Music and its inductive power: A psychobiological and evolutionary approach to musical emotions. *Frontiers in Psychology*, **4**:494.

Reynolds, N. (1989). Opera lovers smell after scratching through Prokofiev. *The Daily Telegraph*, **December 7th**, 3.

Richter, H. (1926). Heizung und Lüftung, Reklamr. In *Das Deutsche Lichtspieltheater in Vergangenheit, Gegenwart Und Zukunft* [The German movie theater in the past, present and future] (pp. 29-32). Berlin, Germany: Prisma-Verlag.

Rimmel, E. (1865). *Rimmel's perfume vaporizer for diffusing the fragrance of flowers in apartments, ball rooms, Etc*. London.

Runciman, J. F. (1915). Noises, smells and colours. *The Musical Quarterly*, **1**, 149-161.

Salesse, R., & Domisseck, S. (2015). Techniques d'odorisation dans les arts vivants. In C. Jaquet (Ed.), *L'art olfactif contemporain* (pp. 79-93). Rencontres 120. Paris: Classiques Garnier.

Sammler, D., Grigutsch, M., Fritz, T., & Koelsch, S. (2007). Music and emotion: Electrophysiological correlates of the processing of pleasant and unpleasant music. *Psychophysiology*, **44**, 293-304.doi:10.1111/j.1469-8986.2007.00497.x

Sanderson, D. (2018). Jazz it up or you'll be history, conductor warns orchestras. *The Times*, **May 7th**, 17.

Schifferstein, H. N. J., Talke, K. S. S., & Oudshoorn, D.-J. (2011). Can ambient scent enhance the nightlife experience? *Chemosensory Perception*, **4**, 55-64.

Schöffer, N. (1985). Sonic and visual structures. *Leonardo*, **18(2)**, 59-68.

Scholes, P. A. (1970). *The Oxford companion to music* (10th Ed.). London, UK: Oxford University Press.

Sebag-Montefiore, C. (2015). The movie you can smell. *BBC Online*, **October 13th**. http://www.bbc.com/culture/story/20151013-the-movie-you-can-smell.

Sebag-Montefiore, C. (2016). Scent of memory: Smell and classical music prove an intoxicating combination. *The Guardian*, **November 11th**. https://www.theguardian.com/music/2016/nov/11/scent-of-memory-smell-and-classical-music-prove-an-intoxicating-combination?CMP=Share_iOSApp_Other.

Sebba, R. (1991). Structural correspondence between music and color. *Color Research & Application*, **16**, 81-88. doi: 10.1002/col.5080160206.

Seo, H.-S., & Hummel, T. (2010) Auditory–olfactory integration: Congruent or pleasant sounds amplify odor pleasantness. *Chemical Senses*, **36**, 301-309

Seo, H.-S., Lohse, F., Luckett, C. R., & Hummel, T. (2014).Congruent sound can modulate odor pleasantness. *Chemical Senses*, **39**, 215-228. doi: 10.1093/chemse/bjt070

Shepherd-Barr, K. (1999). 'Mise en scent': The Théâtre d'Art's *Cantique des cantiques* and the use of smell as a theatrical device. *Theatre Research International*, **24**, 152-159.

Spangenberg, E. R., Grohmann, B., & Sprott, D. E. (2005). It's beginning to smell (and sound) a lot like Christmas: The interactive effects of ambient scent and music in a retail setting. *Journal of Business Research*, **58**, 1583-1589.

Speed, L. J., Atkinson, H., Wnuk, E., & Majid, A. (2021). The sound of smell: Associating odor valence with disgust sounds. *Cognitive Science*, **45**:e12980. DOI: 10.1111/cogs.12980.

Spence, C. (2002). *The ICI report on the secret of the senses*. London: The Communication Group.

Spence, C. (2011). Crossmodal correspondences: A tutorial review. *Attention, Perception, & Psychophysics*, **73**, 971-995.

Spence, C. (2014). Noise and its impact on the perception of food and drink. *Flavour*, **3**:9.

Spence, C. (2015a). Cross-modal perceptual organization. In J. Wagemans (Ed.), *The Oxford handbook of perceptual organization* (pp. 649-664). Oxford, UK: Oxford University Press.

Spence, C. (2015b). Just how much of what we taste derives from the sense of smell? *Flavour*, **4**:30. https://doi.org/10.1186/s13411-015-0040-2.

Spence, C. (2017). *Gastrophysics: The new science of eating*. London, UK: Viking Penguin.

Spence, C. (2019a). Multisensory experiential wine marketing. *Food Quality & Preference*, **71**, 106-116. https://doi.org/10.1016/j.foodqual.2018.06.010.

Spence, C. (2019b). On the relative nature of (pitch-based) crossmodal correspondences. *Multisensory Research*, **32**, 235-265.

Spence, C. (2020a). Assessing the role of emotional mediation in explaining crossmodal correspondences involving musical stimuli. *Multisensory Research,* **33**, 1-29. https://doi.org/10.1163/22134808-20191469.

Spence, C. (2020b). Designing scented colours: On the art & science of olfactory-colour crossmodal correspondences. *The Baltic International Yearbook of Cognition, Logic and Communication*, **14(1)**:2. https://doi.org/10.4148/1944-3676.1125.

Spence, C. (2020c). Scenting the anosmic cube: On the use of ambient scent in the context of the art gallery or museum. *i-Perception,* **11(5)**, 1-26. https://doi.org/10.1177/2041669520966628.

Spence, C. (2020d). Scent and the cinema. *i-Perception,* **11(6)**, 1-22. https://doi.org/10.1177/2041669520969710.

Spence, C. (2020e). Multisensory flavour perception: Blending, mixing, fusion, and pairing within and between the senses. *Foods,* **9(4)**:407. http://dx.doi.org/10.3390/foods9040407.

Spence, C. (2020f). Using ambient scent to enhance well-being in the multisensory built environment. *Frontiers in Psychology* (SI: Smells, Well-being, and the Built Environment), **11**:598859. doi: 10.3389/fpsyg.2020.598859.

Spence, C. (2020g). Olfactory-colour crossmodal correspondences in art, science, & design. *Cognitive Research: Principles & Implications (CRPI),* **5**:52. https://doi.org/10.1186/s41235-020-00246-1. https://rdcu.be/b9oDJ.

Spence, C. (2021a). Scent in the context of live performance. *i-Perception*, **12(1)**:1-28. DOI: 10.1177/2041669520985537.

Spence, C. (2021b). Sonic seasoning and other multisensory influences on the coffee drinking experience. *Frontiers in Computer Science,* **3**:644054. doi: 10.3389/fcomp.2021.644054.

Spence, C. (in press-a). Scent in motion: On the multiple uses of ambient scent in the context of passenger transport. *Frontiers in Psychology*.

Spence, C. (in press-b). Scenting entertainment: Virtual reality storytelling, theme park rides, gambling, & video-gaming. *i-Perception*.

Spence, C., & Deroy, O. (2013). Crossmodal mental imagery. In S. Lacey & R. Lawson (Eds.), *Multisensory imagery: Theory and applications* (pp. 157-183). New York, NY: Springer.

Spence, C., & Gallace, A. (2011a). Multisensory design: Reaching out to touch the consumer. *Psychology & Marketing*, **28**, 267-308.

Spence, C., & Levitan, C. A. (2021). Explaining crossmodal correspondences between colours and tastes. *i-Perception*, **12(2)**:1-28. DOI: 10.1177/20416695211018223.

Spence, C., Ranasinghe, N., Velasco, C., & Obrist, M. (2017). Digitizing the chemical senses: Possibilities & pitfalls. *International Journal of Human-Computer Studies*, **107**, 62-74.

Stevenson, R. J., Rich, A., & Russell, A. (2012). The nature and origin of cross-modal associations to odours. *Perception*, **41**, 606-619. doi:10.1068/p7223 .

Tommasini, A. (2009). Opera to sniff at: A score offers uncommon scents. *The New York Times*, **June 1st**. https://www.nytimes.com/2009/06/02/arts/music/02scen.html.

Uchida, M., Pathak, A., & Motoki, K. (in press). Smelling speech sounds: Association of odours with texture-related ideophones. *Journal of Sensory Studies.*

Velasco, C., Balboa, D., Marmolejo-Ramos, F., & Spence, C. (2014). Crossmodal effect of music and odor pleasantness on olfactory quality perception. *Frontiers in Psychology*, **5**:1352.

von Hornbostel, E. M. (1931). Über Geruchshelligkeit [On smell brightness]. *Pflügers Archiv für die gesamte Physiologie des Menschen und der Tiere*, **227**, 517-538. doi:10.1007/BF01755351

Vuoskoski, J. K., & Eerola, T. (2017). The pleasure evoked by sad music is mediated by feelings of being moved. *Frontiers in Psychology*, **8**:439. doi: 10.3389/fpsyg.2017.00439.

Wasselin, C. (2012). Le concert des parfums [The concert of perfumes]. Downloaded from http://www.michel-godard.fr/download/PDF_INFO/Parfums_French.pdf on 05/01/2013.

Walker-Andrews, A. (1994). Taxonomy for intermodal relations. In D. J. Lewkowicz & R. Lickliter (Eds.), *The development of intersensory perception: Comparative perspectives* (pp. 39-56). Hillsdale, NJ: Lawrence Erlbaum.

Wells, A. (1980). Music and visual color: A proposed correlation. *Leonardo*, **13**, 101-107.

Wesson, D. W., & Wilson, D. A. (2010). Smelling sounds: Olfactory-auditory sensory convergence in the olfactory tubercle. *Journal of Neuroscience*, **30**, 3013-3021.

Wesson, D. W., & Wilson, D. A. (2011). Sniffing out the contributions of the olfactory tubercle to the sense of smell: Hedonics, sensory integration, and more? *Neuroscience and Biobehavioral Reviews*, **35**, 655-668.

White, C. (2011). The smell of commerce: How companies use scents to sell their products. *The Independent*, **August 16th**. http://www.independent.co.uk/news/media/advertising/the-smell-of-commerce-how-companies-use-scents-to-sell-their-products-2338142.html.

Witztum, E., & Lerner, V. (2016). Alexander Nicolaevich Scriabin (1872-1915): Enlightenment or illness? *Journal of Medical Biography*, **24**, 331-338.

Yudov, M. (n.d.). Fragrant notes and redolent sounds. *Fragrantica*. https://www.fragrantica.com/news/Fragrant-Notes-and-Redolent-Sounds-10977.html.

Zhou, C., & Yamanaka, T. (2018). How does congruence of scent and music affect people's emotions. *International Journal of Affective Engineering*, **17(2)**, 127-136. doi: 10.5057/ijae.IJAE-D-17-00032.

Zhou, G., Lane, G., Noto, T., Arabkheradmand, G., Gottfried, J. A., Schuele, S. U., et al. (2019). Human olfactory-auditory integration requires phase synchrony between sensory cortices. *Nature Communications*, **10**, 1-12. doi:10.1038/s41467-019-09091-3

Zimmerman, M. (1989). The nervous system in the context of information theory. In R. F. Schmidt & G. Thews, *Human physiology* (2nd. Complete Ed.) (pp. 166-173). Berlin, Germany: Springer-Verlag.

Figure 1. Scale of crossmodal correspondences between sound and odours reproduced from Piesse (1867, pp. 42-43).

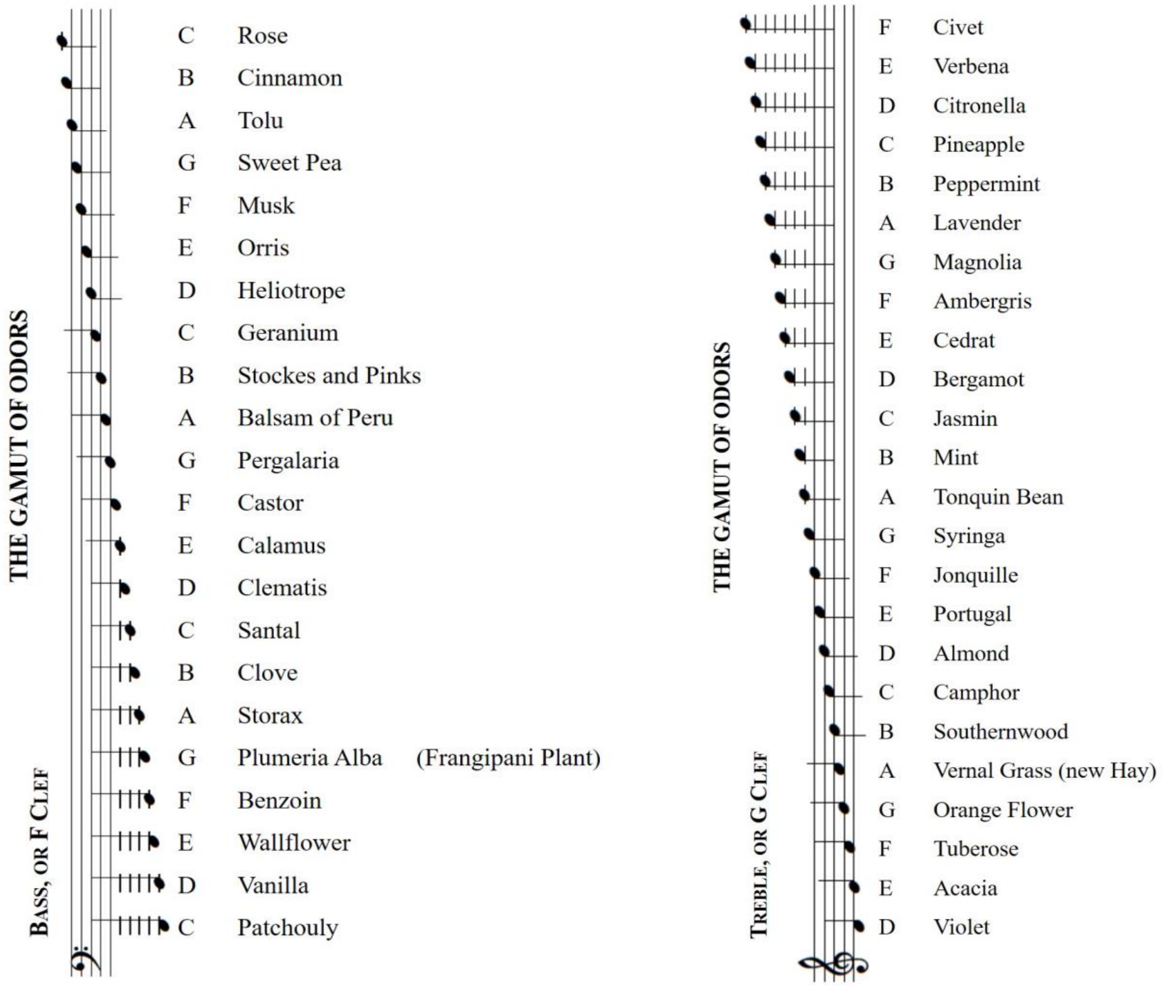

Figure 2. Lee's (2013) mapping of fragrance families to groups of musical notes, used as part of his project "Essence in Space." in which a piece of music is turned into a fragrance.

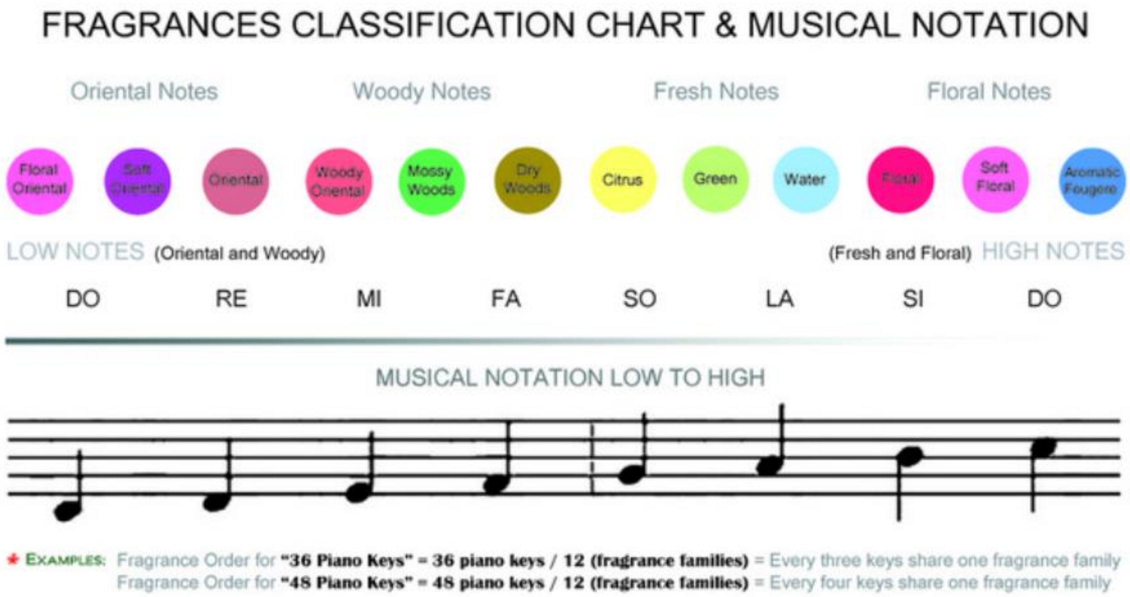

Figure 3. A) Choice of instrument as a function of the odour presented in Crisinel and Spence's (2012a) study in which the participants matched each of 20 typical wine aromas to a specific class of musical instrument. Only those odours that led to a significant preference for a particular class of musical instrument are shown. The total count per category is 30. B) Mean pitch matched to each odour in Crisinel and Spence's (2012a) study. MIDI (musical instrument digital interface) note numbers were used to code the pitch of the chosen notes. Western musical scale notation is shown on the right-hand y-axis. High-pitched notes were preferred for fruits. [Figures reprinted with permission from Crisinel & Spence (2012a).]

A)

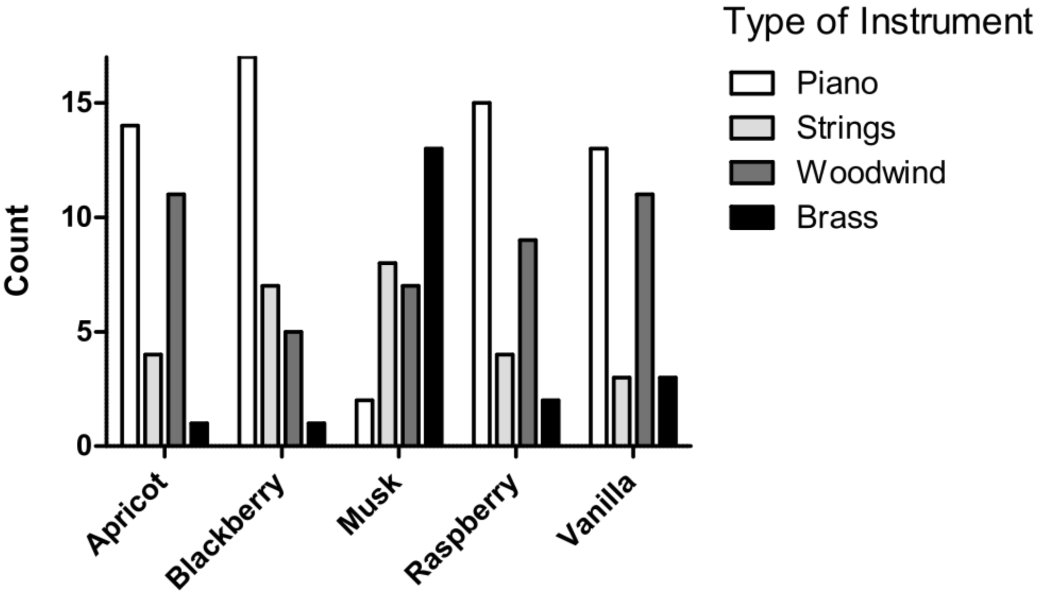

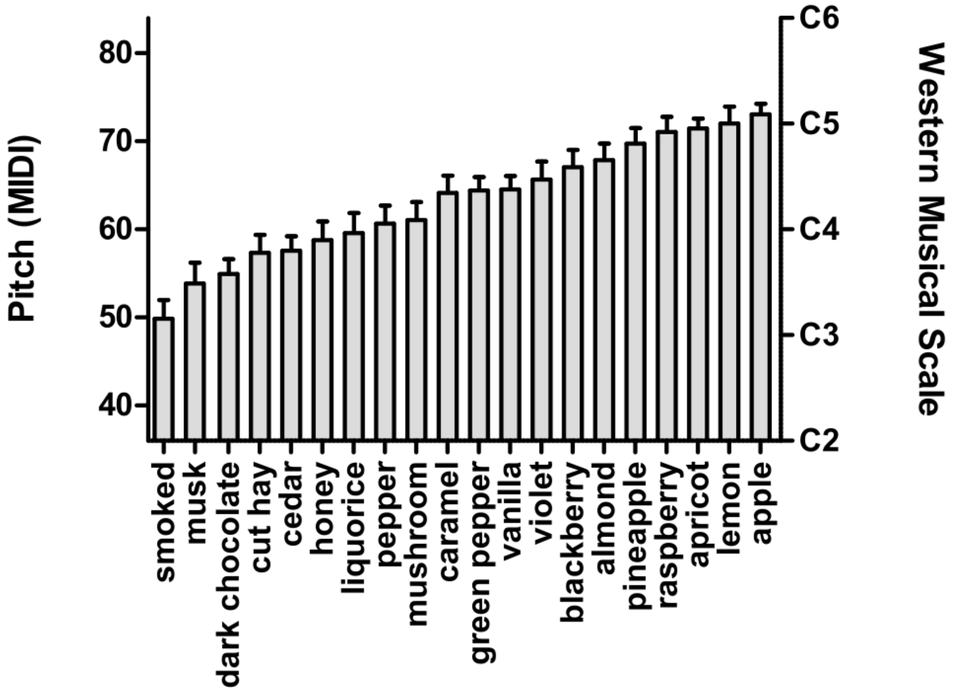

Table 1. Results of Mahdavi et al.’s (2020) study in which 27 people from three countries (Brazil, BR; Iran, IR; and Portugal, PT) were interviewed about their auditory associations (nature sounds, music, and human voice) with a variety of scent descriptors, described verbally. [The number after country code identifies the participant number.]

Scent	Sounds of nature	Music	Human Voice
Sweet scent	N/A	-Ballad music (BR8)<br>-Pop music (IR7)<br>-Classical music (IR3)	N/A
Bitter scent	N/A	-Classical music (IR5)<br>-Heavy metal (IR7)	N/A
Warm scent	-Sound of birds (IR9)	-Spanish flamenco (IR5)<br>-Intimate music (BR1)	-Warm, sensual voice (BR4, BR8, BR9, PT2, PT4, PT6)<br>-Strong voice (PT2, PT8)
Cool scent	-Sound of forest (breeze) (IR9)<br>-Sound of sea (PT4)<br>-Sound of wind (IR8)<br>-Sound of sea waves (IR8, IR4)<br>-Sound of rain (IR8)	N/A	-Soft voice (BR9, IR4, PT8, PT9)
Soft/light scent	-Sound of sea (BR8)<br>-Sound of rain (BR8)	-Calm music (PT1)<br>-Soft music (BR1)<br>-Pop music (BR3)	-Soft voice (BR7, PT5)
Intense/strong scent	N/A	-Intense music (PT1)<br>-Rock music (BR8)<br>-Romantic music (BR7)	-Male voice (PT1)<br>-Mature voice (BR3)<br>-Mature male voice (BR3)
Fresh scent	-Sound of sea (BR7)<br>-Sound of rain (BR7)	N/A	-Young voice (BR3)<br>-Soft voice (BR7)
Floral scent	-Sound of breeze (BR4)	-Soft music (BR5)<br>-Folk music (IR5)	-Soft voice (BR4)
Fruity scent	-Sounds of nature in general (BR4, BR6, BR9)	-Pop music (IR7)	-Soft voice (BR4, BR7)
Woody scent	N/A	-Romantic music (BR4)<br>-Blues music (IR5)<br>-Intimate music (PT4)	-Mature male voice (BR3)
Oriental scent	N/A	-Rock music (IR4)<br>-Intimate music (PT4)<br>-Heavy metal music (IR7)	-Intense voice (PT6)
